# Social jetlag elicits fatty liver via perturbed circulating prolactin rhythm-mediated circadian remodeling of hepatic lipid metabolism

**DOI:** 10.1186/s40779-025-00609-z

**Published:** 2025-06-03

**Authors:** Peng-Zi Zhang, Ying-Huan Shi, Yu-Xin Guo, Ya-Yuan Li, Hong-Li Yin, Tian-Yu Wu, Ye Zhu, Jia-Xuan Jiang, Yan Bi

**Affiliations:** 1 Department of Endocrinology, Endocrine and Metabolic Disease Medical Center, Nanjing Drum Tower Hospital, Affiliated Hospital of Medical School, Nanjing University, Nanjing, 210008 China; 2 Branch of National Clinical Research Centre for Metabolic Diseases, Nanjing, 210008 China; 3 National Key Laboratory for Novel Software Technology and the National Institute of Healthcare Data Science, Nanjing University, Nanjing, 210008 China

**Keywords:** Social jetlag, Prolactin, Circadian rhythm, Metabolic dysfunction-associated steatotic liver disease (MASLD)

## Abstract

**Background:**

The prevalence of circadian misalignment, particularly social jetlag (SJL), contributes significantly to the epidemic of metabolic disorders. However, the precise impact of SJL on the liver has remained poorly elucidated.

**Methods:**

The rhythmicity of circulating prolactin (PRL) was evaluated in subjects with SJL and mice under SJL. The causative mechanism of SJL on fatty liver was explored using jetlag model in wild-type and *Prl*^*−/−*^ mice. Luciferase reporter assay, electrophoretic mobility shift assay, and chromatin immunoprecipitation analysis were used to study the transcriptional mechanism of retinoic acid receptor-related orphan receptor α on PRL. RNA-seq on human and mice liver as well as circadian analysis were used to study the mechanism of SJL-associated desynchronized PRL on hepatic lipid metabolism. The therapeutic effect of PRL intervention on SJL-induced mice at different time points was compared.

**Results:**

SJL increases the risk of metabolic dysfunction-associated steatotic liver disease (MASLD), mediated by the disruption of the rhythmicity of serum PRL. In particular, SJL inhibits the rhythmic transcription of PRL in the pituitary, leading to desynchronized PRL levels in circulation. Under jetlag conditions, the rhythmicity of the hepatic PRL signaling pathway was significantly dampened, which resulted in increased lipogenesis via inhibited hepatic mitogen-activated protein kinase/cyclin D1 expressions. Notably, PRL treatment at PRL nadir in jetlagged mice decreased hepatic lipid content and liver injury markers to a greater extent compared with conventional PRL administration.

**Conclusions:**

Reprogrammed hepatic PRL signaling pathway with concomitant dysregulated lipid metabolism homeostasis was the causative mechanism of fatty liver under SJL, which was mediated through derailed serum PRL rhythm. Restoration of PRL rhythm could effectively alleviate SJL-induced fatty liver, providing new insight into treating MASLD.

**Supplementary Information:**

The online version contains supplementary material available at 10.1186/s40779-025-00609-z.

## Background

With the change in modern lifestyles, metabolic dysfunction-associated steatotic liver disease (MASLD), defined as the steatotic liver disease associated with metabolic syndrome, has become the most common liver disease worldwide with a global prevalence of 32.4% [1]. The growing burden of liver-related and extrahepatic complications highlights the need for preventive approaches.

Beyond overnutrition and sedentariness, circadian misalignment, in which the endogenous clock is desynchronized from environmental cycles, contributes to the onset of fatty liver [2]. In contemporary society, social jetlag (SJL), characterized by the disparity in sleep–wake timing between workdays and free days, represents the prevailing form of chronic circadian misalignment [3]. It is reported that approximately 40–70% worldwide population reported to have experienced SJL for 1 h or more [4, 5]. Noteworthy, SJL is implicated in the increased risk for obesity and type 2 diabetes [6, 7], whereas direct evidence linking SJL to MASLD is lacking. Given the widespread occurrence of SJL and its concurrent metabolic disturbances, it is imperative to assess the correlation between SJL and MASLD.

Rhythmic environmental cues synchronize the master clock in the central nervous system, orchestrating the rhythmic physiological states in peripheral organs via neural and hormonal pathways [8]. Neural regulation is typically rapid in response to stress [9], while hormonal regulation tends to be comparatively slower and more stable, yet whether it can be affected by SJL remains unknown. The pituitary gland, as the master endocrine organ, releases adenohypophyseal hormones essential for growth, reproduction, and metabolism, including growth hormone (GH), prolactin (PRL), thyrotropin-stimulating hormone (TSH), adrenocorticotropic hormone (ACTH), luteinizing hormone (LH), and follicle-stimulating hormone (FSH) [10]. Some hormones, such as GH and PRL, act directly on the liver through specific receptors, while others (TSH, ACTH, LH, and FSH) indirectly stimulate hormone release from secondary endocrine glands [11]. Notably, hormonal secretion exhibits circadian activity, with GH, PRL, TSH, and ACTH displaying typical rhythmicity, while the oscillations of LH and FSH are less well-characterized [12]. The secretion patterns of GH, PRL, and TSH can be disrupted by circadian disturbances induced by external stimuli, such as sleep deprivation [13]. However, whether SJL disrupts hormonal rhythms and their metabolic implications for the liver remains unclear.

In this study, we aimed to analyze the association between SJL and fatty liver using a clinical cohort in which all subjects received liver biopsy. We also explored the role of pituitary hormones and the underlying mechanisms in this process in vitro and in vivo. Finally, we focused on developing an effective therapy strategy for treating SJL-related MASLD.

## Materials and methods

### Patient and public involvement

The clinical cohort study was a case–control study, in which serum PRL levels of control and SJL subjects (*n* = 125 and 72, respectively) from Nanjing Drum Tower Hospital were analyzed. Liver specimens were collected for RNA-sequencing (RNA-seq). The detailed information can be found in Additional file 1: Methods. The protocol of the present study conformed to the guidelines of the Declaration of Helsinki and was approved by the Ethics Committee of Nanjing Drum Tower Hospital, Nanjing University Medical School (2021-388-01). All participants signed informed consent before study inclusion.

### Animals experiments

Female C57BL/6 J (*n* = 130) and *Prl*-knockout (*Prl*^−/−^) mice (*n* = 15) were assigned to normal light cycle and jetlag conditions. All animal studies were conducted in adherence to the guidelines of the Animal Ethics Committee of Nanjing Drum Tower Hospital (2021AE01044). Histopathology, metabolic parameters, and gene expression analysis were performed as described in Additional file 1: Methods.

### Cell and molecular experiments

HepG2, MMQ, and 293 T cells were obtained from the Cell Bank of the Chinese Academy of Sciences. Primary hepatocytes were isolated from female wild-type C57BL/6 or *Prl*^*−/−*^ mice. Detailed methods of cell culture and molecular experiments are given in Additional file 1: Methods.

### RNA-seq

Human and mouse liver tissue were subjected to RNA-seq. The ZeitZeiger algorithm was applied to infer the circadian features of human liver gene expressions [14]. JTK_CYCLE was used to analyze the periodicity of mice liver gene expressions [15]. The details are provided in Additional file 1: Methods.

### Statistical analysis

Clinical data were expressed with mean ± standard deviation (SD) for normally distributed continuous variables or median (interquartile range, IQR) for non-normally distributed variables, with normality assessed using Shapiro–Wilk test; categorical variables are expressed as *n* (%). For animal and cell experiments, data were shown as scatter dot plots with mean. Statistical comparisons employed Student’s *t*-test (parametric) or Mann–Whitney *U* test (non-parametric) for clinical continuous variables, *χ*^2^ test or Fisher’s exact test for categorical data, while experimental data used unpaired two-tailed *t*-tests (two groups) or one-way ANOVA with Tukey’s post hoc test (multiple groups). Advanced analyses [correlation, principal component analysis (PCA), mediation] were performed after verifying model assumptions. All statistical analyses were conducted using SPSS 22.0 and R 4.2.1, with figures generated in GraphPad Prism 9. A two-sided *P* value < 0.05 was considered statistically significant.

## Results

### SJL increases the risk of biopsy-proven MASLD

**Fig. 1:**
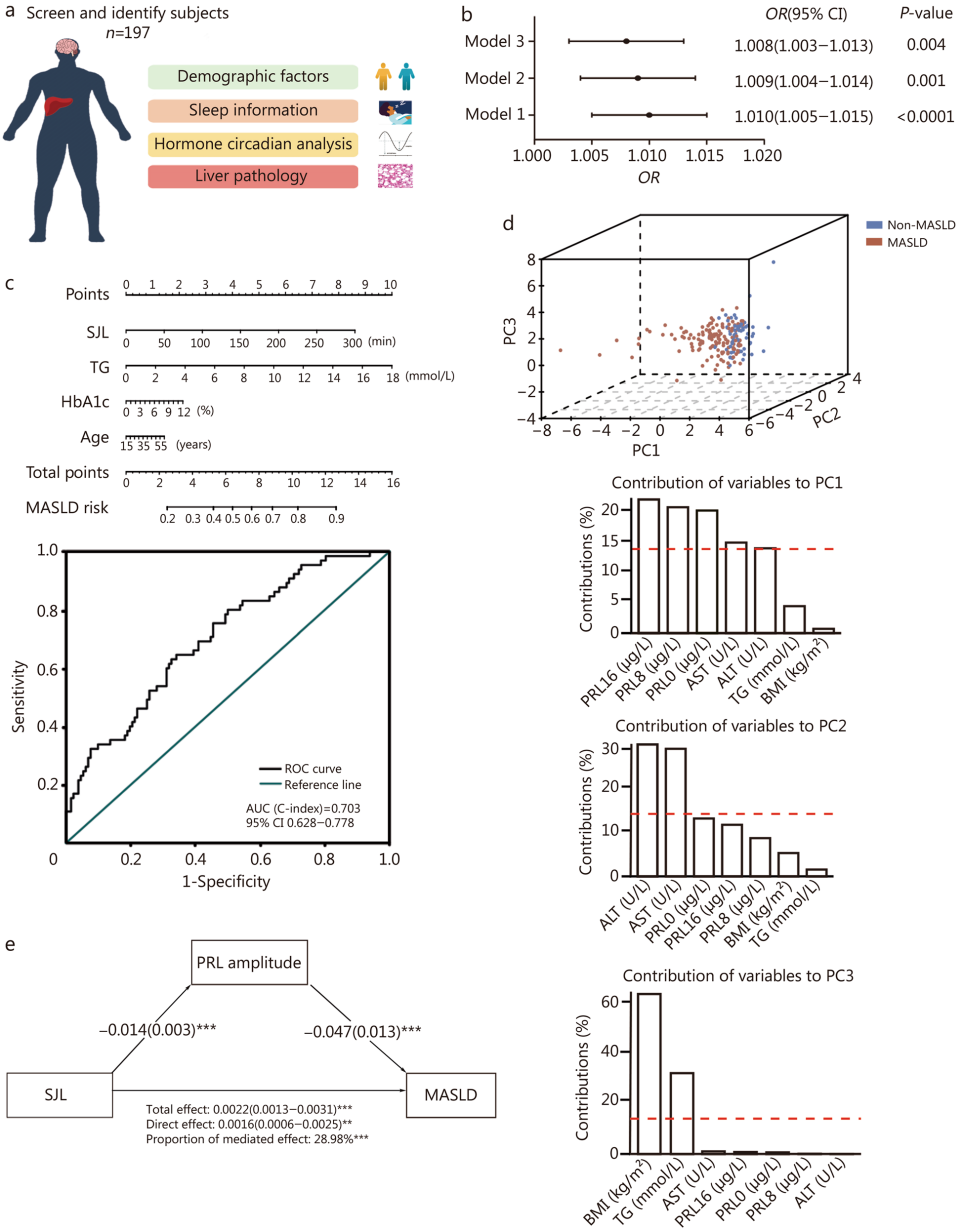
Social jetlag (SJL) induces metabolic dysfunction-associated steatotic liver disease (MASLD), mediated by a disrupted prolactin (PRL) rhythm. **a** Illustration of the procedural workflow in the clinical study. **b** Forest plot of multivariate logistic regression [odds ratio (*OR*) and 95% confidence interval (95% CI)] for SJL and MASLD. Model 1: unadjusted model; Model 2: adjusted for age, sex, and body mass index (BMI); Model 3: adjusted for age, sex, BMI, fasting blood glucose (FBG), alanine aminotransferase (ALT), aspartate aminotransferase (AST), and triglycerides (TG). **c** Nomogram of SJL, TG, hemoglobin A1c (HbA1c), and age to predict MASLD, and receiver operating characteristic (ROC) curve and its diagnostic performance. **d** Principal component analysis (PCA) of metabolic parameters and PRL levels in non-MASLD and MASLD subjects. **e** Mediation analysis of the association between SJL and MASLD, adjusted for age, BMI, and HbA1c. ^*^P < 0.05, ^**^P < 0.01, ^***^P < 0.001

A total of 248 participants who underwent liver biopsy at Nanjing Drum Tower Hospital between January 2019 and November 2021 were recruited. Ultimately, 197 subjects [aged 18–57 years, body mass index (BMI) (29.5 ± 6.5) kg/m^2^] were enrolled to complete the study (Fig. 1a; Additional file 1: Fig. S1a). SJL was measured by subtracting each participant’s midpoint of sleep on work days from their midpoint of sleep on free days. Participants were categorized into control (SJL < 1 h, *n* = 125) and SJL (SJL ≥ 1 h, *n* = 72) groups based on the disparity between the midpoint of weekday and weekend sleep. The SJL group exhibited analogous blood pressure levels but significantly higher hemoglobin A1c (HbA1c), fasting blood glucose (FBG), alanine aminotransferase (ALT), aspartate aminotransferase (AST), and homeostatic model assessment for insulin resistance (HOMA-IR) levels in comparison to the control group. The prevalence of MASLD was significantly higher in the SJL group than in the control group (41.7% vs. 28.0%, *P* < 0.001) (Additional file 1: Table S1). We also analyzed SJL profiles of the study population based on whether they had MASLD or not and found that the percentage of SJL was significantly higher in the MASLD group (Additional file 1: Table S2). Logistic regression analysis revealed associations between SJL and MASLD. Greater SJL values were associated with elevated risk of MASLD (Model 1, unadjusted *OR* = 1.010, *P* < 0.0001), and this association persisted after adjustment for age, sex and BMI (Model 2, *OR* = 1.009, *P* = 0.001), as well as age, sex, BMI, FBG, ALT, AST, and TG (Model 3, *OR* = 1.008, *P* = 0.004) (Fig. 1b). Using variables in Model 3, a nomogram predicting the risk of MASLD was constructed, exhibiting a relatively good discriminative power with an area under the curve (AUC) of 0.703 (95% CI 0.628–0.778; Fig. 1c).

### SJL-induced MASLD is mediated by disturbed PRL rhythm

With respect to the role of pituitary hormones in SJL-associated MASLD, serum PRL, ACTH, TSH, LH, FSH, and GH levels at 8:00, 16:00, and 24:00 were assessed in both the control and SJL groups. PRL secretion in the control group exhibited circadian fluctuations, with the nadir observed in the afternoon and the zenith at 24:00. However, PRL levels at the 3 time points in subjects with SJL ≥ 1 h were remarkably lower than in those with SJL < 1 h [7.48 (5.75, 9.23) μg/L vs. 11.28 (8.53, 14.59) μg/L, 6.74 (5.48, 8.80) μg/L vs. 9.84 (7.47, 14.07) μg/L, 6.82 (5.58, 7.93) μg/L vs. 11.29 (8.81, 16.17) μg/L; *P* < 0.001], while other pituitary hormones at the 3 time points showed no significant differences between the two groups (Additional file 1: Table S1). PRL levels at the 3 time points were inversely associated with the absolute value of SJL (*r* = −0.36, −0.32, −0.47, respectively; *P* < 0.05) and the histopathological degree of steatosis (*r* = −0.29, −0.34, −0.31, respectively; *P* < 0.05). Other hormones exhibited no correlation with either absolute SJL values or steatosis grade (Additional file 1: Fig. S1b). Circadian analysis was further implemented and revealed a significant reduction in the amplitude of PRL in subjects with SJL ≥ 1 h, whereas the amplitude and phase of ACTH, TSH, LH, FSH, and GH were not significantly different between the two groups (Additional file 1: Table S1). The sinus model of serum PRL in control and SJL subjects was fitted using the 3-time point rhythm prediction method with the values for amplitude, mesor, and phase. The equation was *f*(*t*) = 12.55 + 3.55 × sin(x + 0.06) in control subjects, and *f*(*t*) = 7.30 + 1.95 × sin(x − 0.12) in SJL subjects. PCA nalysis based on clinical variables separated MASLD patients from controls, with components 1 (PC1), 2 (PC2), and 3 (PC3) accounting for 77.3% of the variation between samples. PC1, which accounted for the greatest amount of variance (42.1%), included variables of PRL levels from 3-time points, liver enzymes, together with BMI and TG levels (Fig. 1d). Finally, to quantify the effect of PRL rhythm in SJL associated MASLD, mediation analysis was carried out. After adjusting for age, BMI, and HbA1c, mediation analysis showed that SJL-induced MASLD was partially mediated by decreased PRL amplitude, in which the total effect was 0.0022 (95% CI 0.0013–0.0031, *P* < 0.001), and the proportion of mediation effect was 28.98%, *P* < 0.001 (Fig. 1e). These results suggested that the altered PRL rhythm mediated the association between SJL and MASLD.

### Jetlag led to hepatic steatosis and aberrant PRL rhythm in mice

**Fig. 2:**
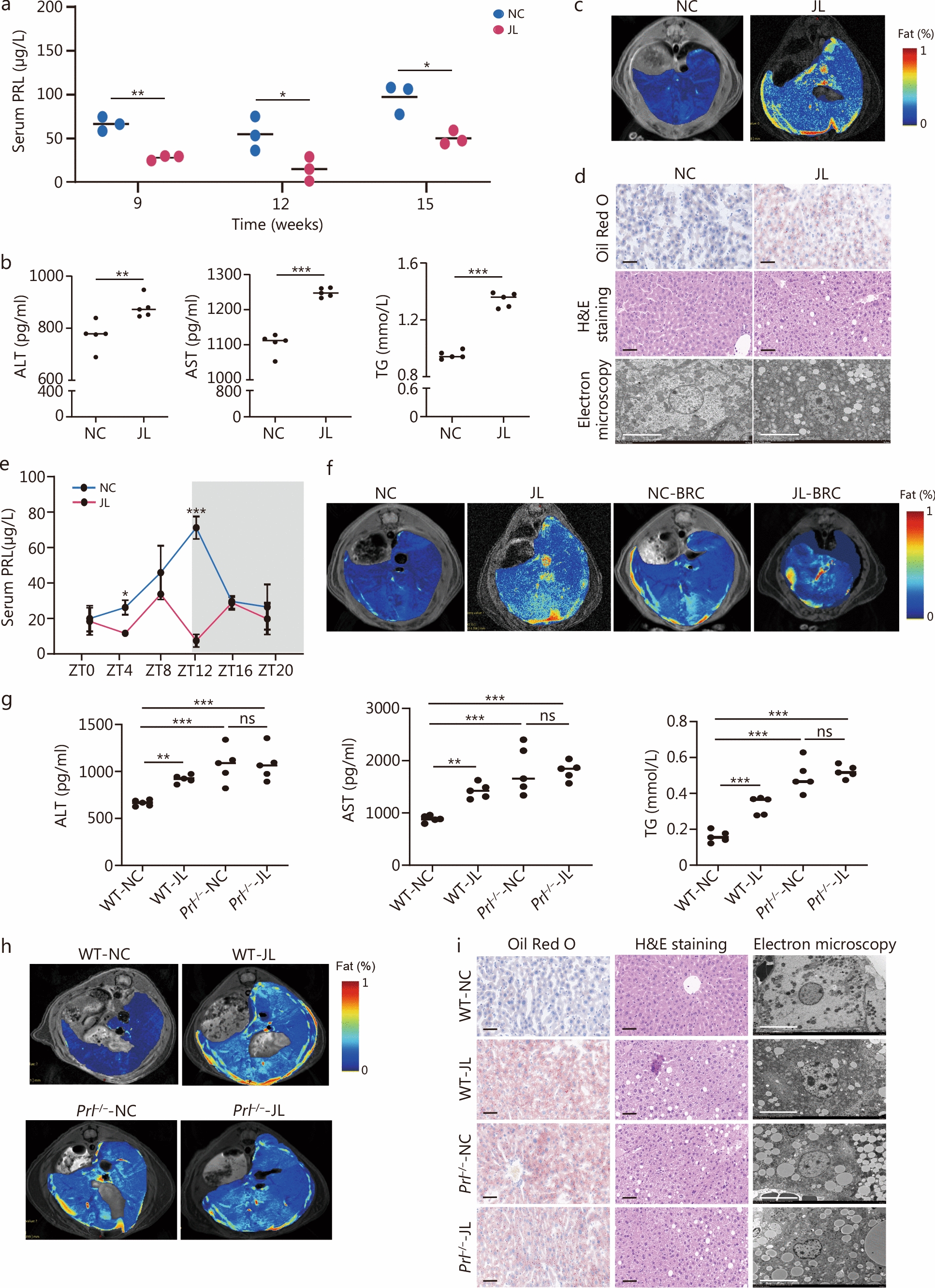
Jetlag led to hepatic steatosis and aberrant prolactin (PRL) rhythm in mice. **a** Serum PRL levels in mice at 9, 12, and 15 weeks of normal light cycle (NC) and jetlag (JL) groups (*n* = 3). **b** Serum levels of alanine aminotransferase (ALT), aspartate aminotransferase (AST), and triglycerides (TG) measured in mice under NC or JL via enzyme-linked immunosorbent assay (ELISA) (*n* = 5). **c** Hepatic lipid content of NC and JL mice measured by magnetic resonance imaging (MRI). **d** Oil Red O staining (scale bar = 50 μm), H&E staining (scale bar = 50 μm), and electron microscopy (scale bar = 10 μm) analysis of lipid content in the liver from mice under NC or JL. **e** Serum levels of pituitary hormone PRL at ZT0, ZT4, ZT8, ZT12, ZT16, and ZT20 from mice under NC or JL. **f** Hepatic lipid content of mice in NC, JL, NC-BRC, and JL-BRC groups was measured by MRI. **g** Serum levels of ALT, AST, and TG in wild-type and *Prl*-knockout mice under NC or JL (*n* = 5). **h** MRI scanning of *Prl-*knockout mice NC or JL. **i** Oil Red O staining (scale bar = 50 μm), H&E staining (scale bar = 50 μm), and electron microscopy (scale bar = 10 μm) analysis of lipid content in the liver from wild-type and *Prl-*knockout mice under NC or JL. ^*^P < 0.05, ^**^P < 0.01, ^***^P < 0.001, ns not significant. *P*-values were calculated by independent-sample *t-*test (**a**, **b**, **e**) and one-way ANOVA (**g**). WT wide-type, BRC bromocriptine mesylate

To induce circadian misalignment, akin to SJL, C57BL/6 mice were subjected to either a normal light cycle (NC group) or jetlag shifts (JL group) for a duration of 16 weeks. Mice in the JL group exhibited similar levels of food intake and body weight (Additional file 1: Fig. S2a, b), higher blood glucose levels at 30 and 120 min during glucose tolerance test (GTT), as well as higher glucose levels at 0, 15, 30, 60, and 120 min during insulin tolerance test (ITT) (Additional file 1: Fig. S2c). Importantly, when dynamically monitored, we observed lower serum PRL levels after 9 weeks (Fig. 2a), and liver steatosis after 15 weeks (Additional file 1: Fig. S2d) of SJL.

At 16 weeks, serum ALT, AST, and TG levels were remarkably elevated in the JL group compared to the NC group (*P* < 0.01, Fig. 2b). Liver magnetic resonance imaging (MRI) scanning documented a higher liver fat fraction in the JL group than the NC group (Fig. 2c). Substantiating these findings, Oil Red O staining, H&E staining, and electron microscopy confirmed greater lipid accumulation in hepatocytes of JL mice relative to NC mice (Fig. 2d). Circadian analysis revealed that in NC mice, serum PRL levels exhibited significant rhythmicity with a peak at ZT12, whereas in JL group mice, PRL levels were reduced at all time points, particularly at ZT12 (*P* < 0.001, Fig. 2e). Importantly, using the CircaCompare algorithm, rhythmicity test showed that in mice under NC conditions, serum PRL levels oscillated strikingly during 24 h (*P* < 0.05), however, this rhythmicity was significantly dampened in JL mice (*P* > 0.05; Additional file 1: Fig. S2e). These results suggested that jetlag induced fatty liver and disrupted the rhythmic secretion of PRL in mice. We also examined hepatic mRNA levels of key enzymes involved in hepatic lipogenesis such as *Cidea*, fatty acid synthase (*Fasn*), *Pparγ*, and *Acc*. We found that gene expressions of *Cidea*, *Fasn*, and *Acc* were remarkably increased in the liver of JL mice, while *Pparγ* expressions were not statistically different between the two groups (Additional file 1: Fig. S2f).

To disrupt endogenous PRL secretion, C57BL/6 mice under normal light cycle (NC) or jetlag shifts conditions (JL) were intraperitoneally injected with PRL inhibitor bromocriptine mesylate (BRC, 5 mg/kg) daily under (NC-BRC) and for 16 weeks. The results indicated that both NC-BRC mice and JL-BRC mice developed mild hepatic steatosis compared to NC mice, yet NC-BRC and JL-BRC mice showed similar extent of liver steatosis (Fig. 2f).

To further explore the role of PRL in maintaining liver homeostasis, global *Prl*^−/−^ mice were generated. Compared with wild-type mice, *Prl*^−/−^ mice exhibited increased glucose levels at 0, 30, and 60 min during GTT, and higher concentrations of glucose at 0, 15, and 120 min during ITT (Additional file 1: Fig. S2g). Then *Prl*^−/−^ mice were subjected to a normal light cycle (*Prl*^−/−^-NC) or jetlag shifts (*Prl*^−/−^-JL) for 16 weeks. *Prl*^−/−^ mice exhibited increased serum ALT, AST, and TG levels (Fig. 2g), higher liver fat content as assessed by MRI (Fig. 2h) and histological analysis (Fig. 2i), relative to wild-type mice under a normal light cycle (WT-NC). Meanwhile, *Prl*^−/−^-JL mice exhibited similar serum ALT, AST, and TG levels (Fig. 2g), as well as hepatic lipid content relative to *Prl*^−/−^-NC mice (Fig. 2h, i). These findings suggested that jetlad did not aggravate hepatic steatosis in the absence of PRL.

### Jetlag perturbs PRL rhythm by impeding retinoic acid receptor-related orphan receptor α (RORα)/ROR response element (RORE) binding of *Prl* promoter

**Fig. 3:**
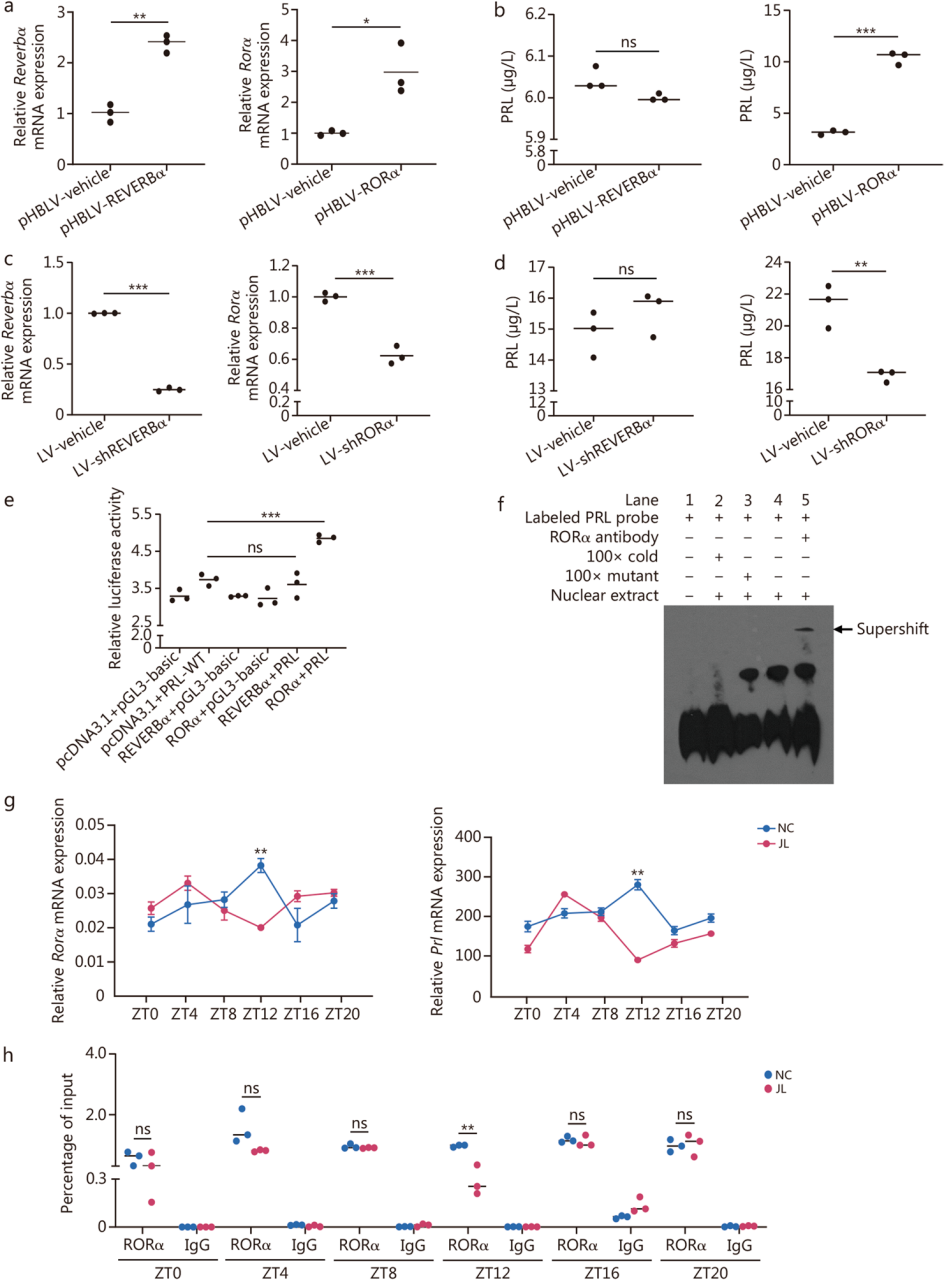
Jetlag perturbs prolactin (PRL) rhythm by impeding retinoic acid receptor-related orphan receptor α (RORα)/ROR response element (RORE) binding of *Prl* promoter. **a** mRNA levels of nuclear receptor subfamily 1 group D member 1 (*Reverbα*) and *Rorα* in pHBLV-REVERBα and pHBLV-RORα MMQ cell lines, respectively (*n* = 3). **b** PRL content in supernatants from pHBLV-REVERBα and pHBLV-RORα MMQ cell lines (*n* = 3). **c** mRNA levels of *Reverbα* and *Rorα* in MMQ cell lines transfected with LV-shREVERBα and LV-shRORα and their respective control, respectively (*n* = 3). **d** PRL content in supernatants of MMQ cell lines transfected with LV-shREVERBα or LV-shRORα (*n* = 3). **e** Effect of REVERBα and RORα on the luciferase activity of WT-*Prl* reporter systems measured by luciferase reporter assay in 293 T cells (*n* = 3). **f** Electrophoretic mobility shift analysis (EMSA) of RORα DNA-binding activity in pituitary extracts prepared from the nonfunctional human pituitary tumor. **g** qRT-PCR analysis of mRNA levels of *Rorα* and *Prl* in the pituitary tissue from mice in the normal light cycle (NC) and jetlag (JL) group at different time points ZT0 (08:00), ZT4 (12:00), ZT8 (16:00), ZT12 (20:00), ZT16 (24:00), ZT20 (04:00) (*n* = 3). **h** ChIP analysis of the pituitary tissue from mice in the NC and JL group at different time points ZT0, ZT4, ZT8, ZT12, ZT16, and ZT20. IgG was used as the negative control, and the product was analyzed by qPCR. ^*^P < 0.05, ^**^P < 0.01, ^***^P < 0.001, ns not significant. *P*-values were calculated by independent-sample *t*-test (**a**, **b**, **c**, **d**, **g**, **h**) and one-way ANOVA (**e**). ANOVA analysis of variance

Subsequently, we sought to elucidate the mechanisms underlying the disruption of PRL oscillation in jetlag. In mammals, the rhythmic expression of genes is orchestrated by a transcriptional feedback loop involving clock genes such as *Rorα* and nuclear receptor subfamily 1 group D member 1 (*Reverbα*) [16]. Sequence analysis revealed a highly conserved DNA-binding motif RORE (5′-AGGTCA-3′) in *Prl* promoters from mice, rats, and humans, as opposed to the E-box (Additional file 1: Fig. S3a). Given that RORE serves as a binding site for transcription factors, REVERBα and RORα, we established rat prolactinoma MMQ cell lines with overexpression of these transcription factors (Additional file 1: Fig. S3b, c). The mRNA levels of *Reverbα* and *Rorα* were significantly up-regulated in pHBLV-REVERBα and pHBLV-RORα MMQ cell lines, respectively, compared with their respective controls (Fig. 3a). Additionally, a markedly higher PRL content was observed in the supernatants of MMQ cells overexpressing RORα but not REVERBα (Fig. 3b). Then lentivirus short hairpin RNA (shRNA) against REVERBα (LV-shREVERBα) and RORα (LV-shRORα) were constructed (Additional file 1: Fig. S3d) and transfected into MMQ cell lines (Additional file 1: Fig. S3e), in which mRNA levels of *Reverbα* and *Rorα* were significantly decreased compared with their respective vehicle (Fig. 3c). As expected, PRL levels in the supernatants of MMQ cells that transfected with lentivirus-mediated shRNA targeting RORα were significantly decreased compared with vehicle lentivirus transfection (LV-vehicle), while did not significantly altered after LV-shREVERBα transfection (Fig. 3d). Subsequently, 293 T cells were transfected with REVERBα and RORα for luciferase reporter gene assay (Additional file 1: Fig. S3f). RORα transfection resulted in a 1.3-fold increase in luciferase activity compared with pcDNA3.1 empty vector transfection whereas overexpression of REVERBα did not influence luciferase activity (Fig. 3e). These findings suggested that RORα, but not REVERBα, transcriptionally activated the *Prl* promoter.

To delineate the binding site of RORα on the *Prl* promoter, an electrophoretic mobility shift assay (EMSA) was conducted using human nonfunctional pituitary adenoma, which was diagnosed based on normal hormone levels and negative immunohistochemical staining. Labeled PRL probe was used as negative control (lane 1), and specific binding of nuclear extract to the wild-type probe (RORE, 5’-AGGTCA-3’) was observed (lane 4). Moreover, a 100-fold excess of unlabeled wild-type probe competitively inhibited binding (lane 2), whereas unlabeled probes with mutated RORE failed to compete with this binding (lane 3) (Fig. 3f). Next, qRT-PCR analysis revealed that the circadian pattern of *Rorα* and *Prl* mRNA expression in the pituitary tissue of C57BL/6 mice synchronized, exhibiting a circadian surge at ZT12. However, jetlag significantly suppressed *Rorα* and *Prl* mRNA expression at ZT12 (Fig. 3g). Finally, we sought to evaluate the effect of SJL on the binding between RORα and *Prl* promoter region using ChIP-qPCR assay in NC and JL mice pituitary tissues. We observed that RORα was recruited to the *Prl* promoter at all time points in pituitary tissues from NC mice, however, a significant reduction in this recruitment was observed in the JL group at ZT12 (Fig. 3h). These data suggest that *Prl* expression is transcriptionally regulated by RORα, a process that is compromised by jetlag.

### SJL alters the PRL signaling pathway and rhythmicity of gene features in human livers

To comprehend the molecular profiles implicated in SJL-associated MASLD, we conducted RNA-seq analysis on liver tissues of 197 subjects. We employed two distinct strategies to analyze this dataset. The first strategy was to analyze differential expression genes (DEGs) of 197 subjects (125 control and 72 SJL subjects), then the DEGs were subjected to Kyoto Encyclopedia of Genes and Genomes (KEGG) analysis. The second strategy involved using the ZeitZeiger algorithm [14], a novel machine-learning tool, to predict the time of gene expression data in control and SJL subjects.

**Fig. 4:**
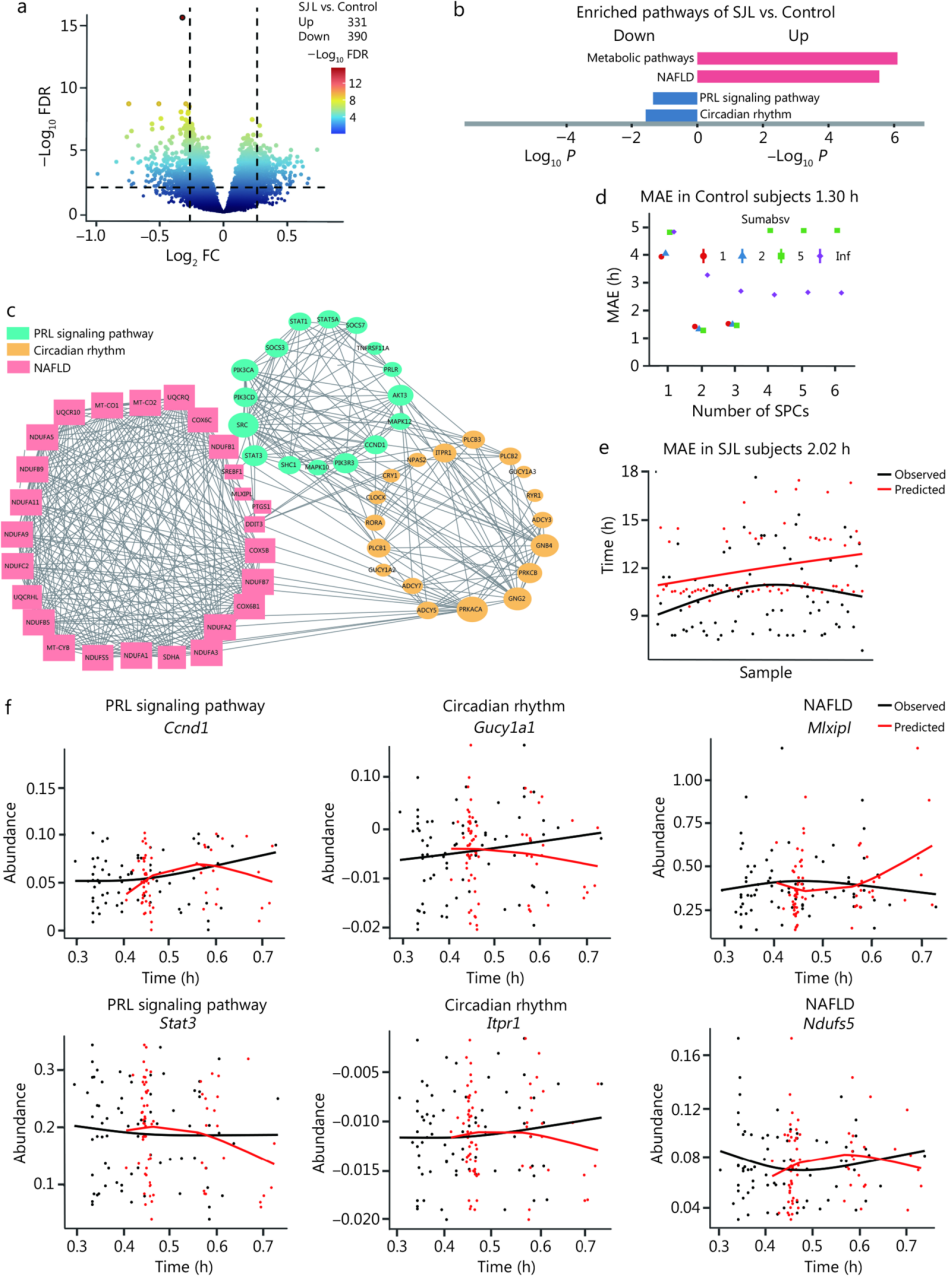
Social jetlag (SJL) alters the prolactin (PRL) signaling pathway and rhythmicity of gene features in human livers. **a** Volcano plot of differential gene expression analysis in the liver between control (*n* = 125) and SJL subjects (*n* = 72). **b** Pathways involved in the PRL signaling pathway, circadian rhythm, and non-alcoholic fatty liver disease (NAFLD) that were annotated based on differential expression genes (DEGs) from SJL vs. control subjects. **c** STRING analysis of DEGs involved in the PRL signaling pathway, circadian rhythm, and non-alcoholic fatty liver disease (NAFLD). **d** Mean absolute error (MAE) on cross-validation using sumabsv (sumabsv = 2) and sparse principal component (SPC = 2), sumabsv means regularization parameter and Inf indicates infinite. **e** ZeitZeiger analysis of the rhythmic curve of the hepatic transcriptome in SJL subjects. **f** ZeitZeiger analysis of the rhythmic curve of canonical genes regarding the PRL signaling pathway, circadian rhythm, and NAFLD in SJL subjects. FDR false discovery rate, Ccnd1 cyclin D1, Gucy1a1 guanylate cyclase 1 soluble alpha 1, Mlxipl MLX interacting protein-like, Stat3 signal transducer and activator of transcription 3, Itpr1 inositol 1,4,5-trisphosphate receptor type 1, Ndufs5 NADH ubiquinone oxidoreductase subunit S5

A total of 721 DEGs were identified between the control and SJL groups (331 up-regulated and 390 down-regulated) (Fig. 4a). These DEGs were annotated to the KEGG pathway database, which included the PRL signaling pathway, circadian rhythm, and NAFLD (Fig. 4b). However, pathways related to TSH receptor, LH receptor, FSH receptor, GH receptor, and glucocorticoid receptor did not exhibit significant enrichment of DEGs between SJL and control subjects (Additional file 1: Fig. S4). Significantly, STRING analysis revealed a close interconnection among genes from these identified pathways (Fig. 4c).

Next, the ZeitZeiger algorithm was employed to analyze RNA-seq data from 125 control and 72 SJL subjects. This machine learning approach derives predictors that map gene abundance to observed time, representing the real-time of receiving liver biopsy [14]. Subsequently, the liver gene expression of subjects in the control group was used as the training dataset to acquire predictors. To assess the accuracy of the predictors, the mean absolute error (MAE) was used, which represents the mean of the differences between the observed and predicted time of each sample. The MAE was 1.30 h in control subjects, indicating relatively accurate prediction performance in the training dataset (Fig. 4d). However, the MAE was 2.02 h in SJL subjects (testing dataset) (Fig. 4e), indicating that the predicted internal time of the gene expression pattern in the liver was different from the observed time in SJL subjects. Specifically, with respect to canonical genes involved in pathways of PRL signaling, circadian rhythm, and NAFLD, the fitted curve of the predicted time and the observed time demonstrated that the oscillation of gene expression in these pathways was altered in the liver of SJL subjects (Fig. 4f).

### Jetlag alters the circadian transcriptome in the liver of mice

**Fig. 5:**
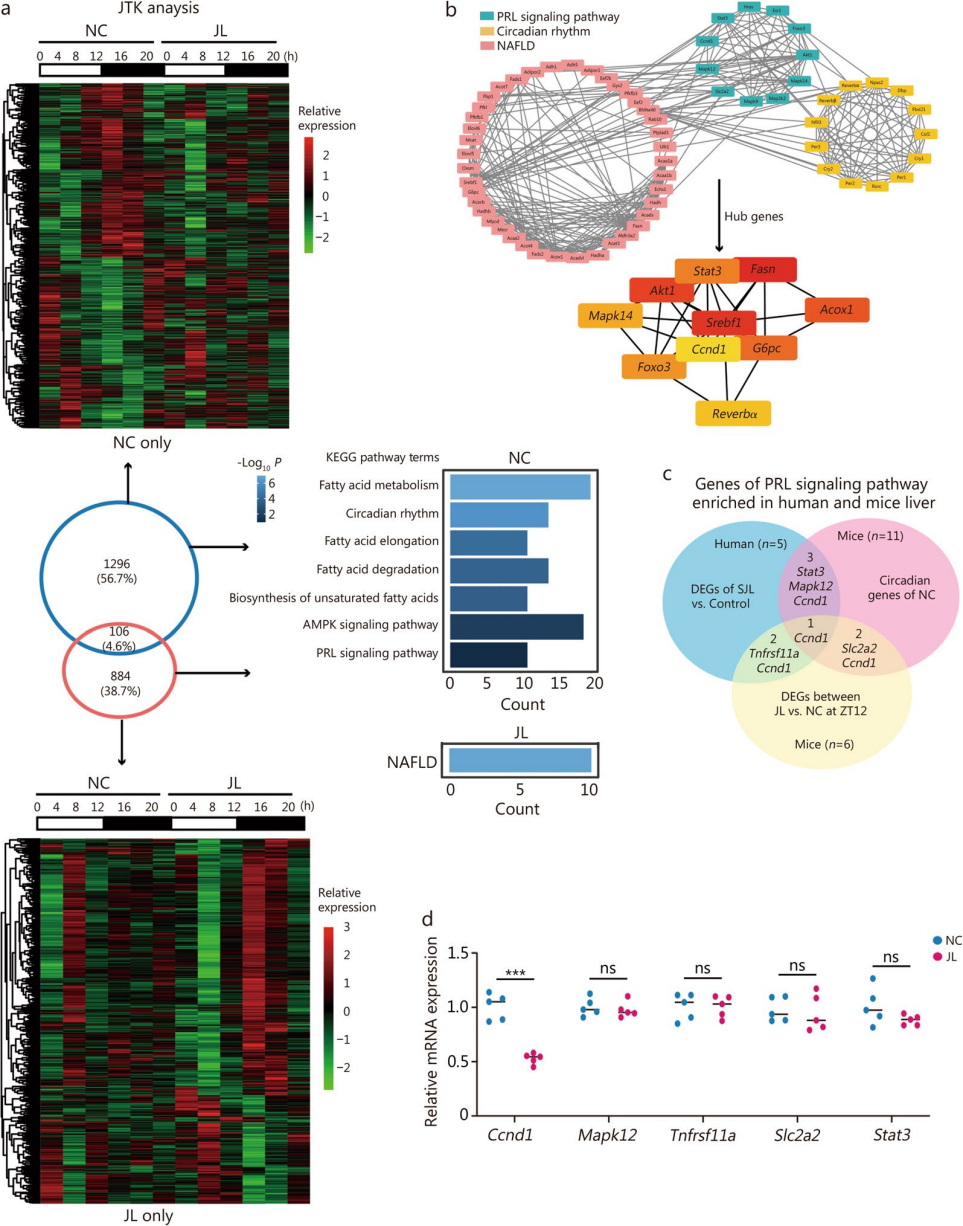
Jetlag reprograms the circadian transcriptome in the liver of mice. **a** Differential gene heat map of transcriptome sequencing in the liver tissue of mice under normal light cycle (NC) and jetlag (JL) at each time point (*n* = 3 of each group per time point). The color bar indicates the scale used to show the expression of the gene across 6-time points, with the highest expression normalized to 1. The number of specific circadian gene transcription in NC and JL conditions was identified as *P* < 0.05 via JTK_CYCLE (upper and down panel). Kyoto Encyclopedia of Genes and Genomes (KEGG) enrichment of specific circadian gene transcription in NC and JL conditions (middle panel). **b** Analysis of protein–protein interaction (PPI) networks within the PRL signaling pathway, circadian rhythm, and lipid metabolism. Hub genes were identified in Cytoscape with the plugin of CytoHubba. **c** Venn diagram illustrating annotated genes commonly enriched in the PRL signaling pathway in both human and mouse liver transcriptome datasets. **d** qRT-PCR validation of liver mRNA levels of genes involved in the PRL signaling pathway of NC and JL mice, data were presented as scatter dot plots with mean and normalized to *β-actin* mRNA levels (*n* = 5). ^***^P < 0.001, ns not significant. *P*-values were calculated by independent-sample *t-*test. PRL prolactin, NAFLD non-alcoholic fatty liver disease, AMPK AMP-activated protein kinase, DEGs differentially expressed genes, Acox1 acyl-coenzyme A oxidase 1, Srebf1 sterol regulatory element binding transcription factor 1, G6pc glucose-6-phosphatase catalytic, *Stat3* signal transducer and activator of transcription 3, Fasn fatty acid synthase, Reverbα nuclear receptor subfamily 1 group D member 1, Foxo3 forkhead box O3, Mapk14 mitogen-activated protein kinase14, Akt1 serine/threonine kinase 1, Mapk12 mitogen-activated protein kinase12, *Ccnd1* cyclin D1, Tnfrsf11a tumor necrosis factor receptor superfamily member 11a, Slc2a2 solute carrier family 2 member 2

Furthermore, we conducted RNA-seq analysis on liver tissues from mice subjected to either a normal light cycle (NC) or jetlag (JL) at 4-h intervals to elucidate how jetlag modulates liver metabolic pathways. Rhythmic transcripts were identified using the JTK_cycle algorithm in the NC group. A total of 1296 genes exhibited oscillation exclusively in the liver of the NC group (Fig. 5a). These oscillatory genes in the NC group, annotated for KEGG analysis, were predominantly enriched in pathways related to circadian rhythm, lipid metabolism processes (including fatty acid metabolism, fatty acid degradation, fatty acid elongation, AMPK signaling pathway, and biosynthesis of unsaturated fatty acids), and the PRL signaling pathway (*P* < 0.05; Fig. 5a, Additional file 1: Fig. S5).

Subsequently, microarray data from liver tissues of male mice maintained in a normal light cycle were analyzed using the National Center for Biotechnology Information (NCBI) Gene Expression Omnibus (GEO) database (GSE52333) [17]. Processed with the JTK algorithm, rhythmic genes were found to be enriched in the PRL signaling pathway, circadian rhythm, and lipid metabolism processes (including fatty acid biosynthesis, AMPK signaling pathway, peroxisome-proliferator-activated receptors signaling pathway) (*P* < 0.05; Additional file 1: Fig. S6). Additionally, we examined the rhythmicity of downstream pathways of other pituitary hormones in the liver from our data and GSE52333. The results indicated that the TSH receptor, LH receptor, FSH receptor, GH receptor, and glucocorticoid receptor pathways did not significantly oscillate in the liver in both datasets (Additional file 1: Figs. S5, S6).

Finally, we observed a substantial change in circadian gene expression profiles in JL mice livers compared to NC. A total of 884 genes exhibited oscillation exclusively in the liver of the JL group. Moreover, the circadian rhythmicity of genes involved in circadian rhythm, PRL signaling pathway, and lipid metabolism processes (including fatty acid biosynthesis, AMPK signaling pathway, peroxisome-proliferator-activated receptors signaling pathway) was significantly attenuated (Rhythmicity test *P* > 0.05), while genes associated with NAFLD gained oscillation and were significantly enriched (*P* = 0.01; Fig. 5a, Additional file 1: Fig. S7). These results confirmed the rhythmicity of genes involved in circadian rhythm, PRL signaling pathway, and lipid metabolism processes, while jetlag dampened cyclic levels of these genes and induced periodic expression of genes linked with MASLD.

To illustrate the association between the 3 common oscillating pathways mentioned under NC conditions, enriched genes from these transcripts (8 genes from the PRL signaling pathway, 13 genes from circadian rhythm, and 36 genes from lipid metabolism processes) were imported into the STRING database to obtain the protein–protein interaction (PPI) network. The PPI network, comprising 57 nodes and 272 edges, demonstrated that PRL signaling was highly interconnected with circadian rhythm and lipid metabolism (*P* < 1 × 10^–16^). Next, these genes were imported into Cytoscape software and clustered into functional communities. Subsequently, 3 clusters of oscillating pathways were identified with a default cutoff on the closeness CytoHubba. The top 10 identified hub genes were *Reverbα* from circadian rhythm, and *Fasn*, acyl-coenzyme A oxidase 1 (*Acox1*), sterol regulatory element binding transcription factor 1 (*Srebf1*), and glucose-6-phosphatase catalytic (*G6pc*) from lipid metabolism, as well as serine/threonine kinase 1 (*Akt1*), cyclin D1 (*Ccnd1*), forkhead box O3 (*Foxo3*), signal transducer and activator of transcription 3 (*Stat3*), and mitogen-activated protein kinase14 (*Mapk14*) from the PRL signaling pathway (Fig. 5b).

Overall, the hepatic PRL signaling pathway played a crucial physiologic role in bridging circadian rhythm and lipid metabolism under normal light cycle; while under jetlag, genes involved in the “NAFLD” pathway were highly enriched.

### Suppressed PRL signaling pathway under jetlag leads to increased lipid synthesis

As serum PRL levels were notably suppressed at ZT12, we further analyzed the RNA-seq data from the livers of NC and JL mice at ZT12. A total of 855 DEGs were identified, consisting of 562 up-regulated and 293 down-regulated genes between the NC and JL groups. With a significance level of *P* < 0.05, up-regulated DEGs were enriched in 18 KEGG pathways, including pathways related to NAFLD and circadian rhythm. Down-regulated DEGs were enriched in 45 pathways, including those related to the PRL signaling pathway and circadian rhythm (Additional file 1: Figs. S8, S9a, b). After that, the mechanisms involved in the desynchronization of hepatic PRL signaling in SJL-induced fatty liver were explored. Pathways of the PRL signaling pathway and circadian rhythm were significantly enriched in both human and mouse transcriptomes and were further analyzed. Overlapping genes that were commonly annotated in the PRL signaling pathway of both human and mouse datasets were identified: *Stat3*, *Mapk12*, *Ccnd1*, *Tnfrsf11a*, and *Slc2a2* (Fig. 5c).

**Fig. 6:**
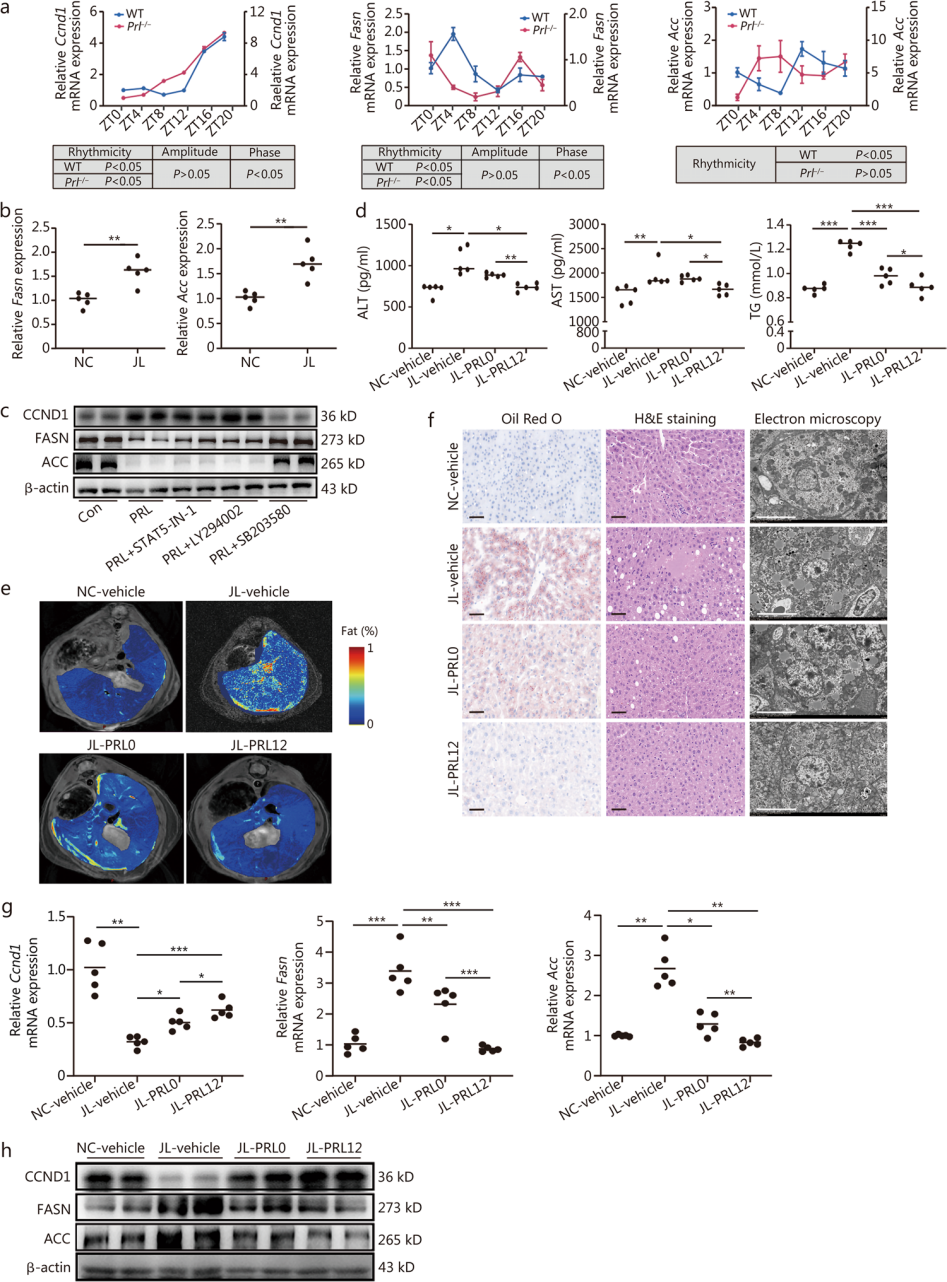
Suppressed PRL signaling pathway under jetlag leads to increased lipid synthesis, and rhythmic administration of PRL rescues hepatic steatosis in mice under jetlag. **a** qRT-PCR analysis of mRNA levels of genes involved in the PRL signaling pathway and lipid metabolism from primary hepatocytes in wild-type (WT) and *Prl*^−/−^ mice after serum shock at indicated time points. The rhythmicity of genes in primary hepatocytes was analyzed using the CircaCompare algorithm, *P* < 0.05 was considered rhythmic. In terms of rhythmic genes, amplitude and phase were further compared between groups. **b** qRT-PCR analysis of mRNA levels of *Fasn* and *Acc* in the normal light cycle (NC) and jetlag (JL) mice (*n* = 5). **c** Western blotting analysis of CCND1, FASN, and *Acc* in HepG2 cells, treated with PRL, LY294002, STAT5 inhibitor-1 (STAT5-IN-1) and SB203580; β-actin was used as an internal reference. **d** Enzyme-linked immunosorbent assay (ELISA) analysis of serum levels of alanine aminotransferase (ALT), aspartate aminotransferase (AST), and triglycerides (TG) in mice from NC-vehicle, JL-vehicle, JL-PRL0, and JL-PRL12 groups (*n* = 5). **e** Liver magnetic resonance imaging (MRI) analysis of hepatic lipid content of mice from NC-vehicle, JL-vehicle, JL-PRL0, and JL-PRL12 groups (*n* = 3). **f** Oil Red O staining (scale bar = 50 μm), H&E staining (scale bar = 50 μm), and electron microscopy (scale bar = 10 μm) analysis of lipid content in the liver from mice in NC-vehicle, JL-vehicle, JL-PRL0, and JL-PRL12 groups. **g** qRT-PCR of *Ccnd1*, *Fasn*, and *Acc* levels in the liver of mice from NC-vehicle, JL-vehicle, JL-PRL0, and JL-PRL12 groups. Data were normalized to *β-actin* mRNA levels. **h** Western blotting analysis of CCND1, FASN, and ACC in the liver of mice from NC-vehicle, JL-vehicle, JL-PRL0, and JL-PRL12 groups. ^*^P < 0.05, ^**^P < 0.01, ^***^P < 0.001. *P*-values were calculated by independent-sample *t-*test (**b**) and one-way ANOVA test (d, g, in terms of ALT in Fig. 6d and *Ccnd1* in Fig. 6g, Welch’s *F*-test was used together with the Games-Howell test for post hoc comparisons). PRL prolactin, CCND1 cyclin D1, FASN fatty acid synthase, ACC acetyl coenzyme, ANOVA analysis of variance

Independent qRT-PCR validation analysis revealed that only *Ccnd1* was significantly down-regulated in the JL group compared with NC mice at ZT12, while other genes were not significantly altered (Fig. 5d). CCND1 is a key cell cycle protein that could repress expressions of de novo lipogenic enzymes in the liver, including FASN and acetyl-CoA carboxylase (ACC) [18]. CCND1, FASN, and ACC were rhythmically expressed in the liver. In this regard, primary mouse hepatocytes from wild-type and *Prl*^−/−^ mice were serum shocked to synchronize the circadian clock to determine whether the circadian expression of these genes was affected by PRL. A loss of rhythmicity was observed in *Ccnd1*, *Fasn*, and *Acc* expression in hepatocytes of *Prl*^−/−^ mice compared with those in wild-type mice (Fig. 6a). Moreover, hepatic mRNA levels of *Fasn* and *Acc* were remarkably increased in JL mice at ZT12 (Fig. 6b). In HepG2 cells, we found that PRL enhanced protein levels of CCND1, which were significantly blocked by SB203580 (MAPK inhibitor) but not LY294002 (PI3K/Akt inhibitor) and STAT5-IN-1 (STAT5 inhibitor) (Fig. 6c). These data suggested that the defect of PRL rhythm in response to SJL inhibit hepatic CCND1 levels through MAPKs, and then enhanced the expression of hepatic lipogenic genes in the liver*.* In addition, DEGs of NC and JL mice liver that annotated to the circadian rhythm at ZT12 (*Per3*, *Reverbα*, *Reverbβ,* and *Rorc*) were also validated. qRT-PCR analysis uncovered that mRNA levels of *Per3* and *Reverbα* were significantly down-regulated while *Rorc* was significantly up-regulated relative to NC mice. In contrast, *Reverbβ* was similar between the two groups at ZT12 (Additional file 1: Fig. S9c).

### Administration of PRL at nadir rescues hepatic steatosis in jetlagged mice

To elucidate the therapeutic impact of PRL rhythmicity on jetlag-induced hepatic steatosis, C57BL/6 mice were categorized into the normal light cycle (NC-vehicle) and jetlag shifts (JL-vehicle) groups for 16 weeks. Subsequently, mice in the JL group were intraperitoneally administered PRL (1 mg/kg) (JL-PRL) at ZT0 (JL-PRL0) or ZT12 (JL-PRL12) for 2 weeks. In GTT, glucose levels at each time point were significantly increased in JL mice compared to NC mice, in which glucose levels of JL mice at 0, 15, 30, and 120 min were significantly restored in JL-PRL0 mice, and were significantly restored in JL-PRL12 mice at each time point. At 15, 30, 60, and 120 min, JL mice that received PRL treatment at ZT12 exhibited remarkably decreased glucose levels than mice that received PRL treatment at ZT0. During ITT, JL mice displayed a marked increment of glucose levels across 5 time points than NC mice. PRL intervention at ZT0 significantly decreased glucose levels at 0, 60, and 120 min of JL mice, and PRL intervention at ZT12 significantly decreased glucose levels at 0, 30, 60, and 120 min of JL mice. Compared with JL-PRL0 mice, JL-PRL12 mice showed a pronounced reduction of glucose levels at 0 and 120 min (Additional file 1: Fig. S9d). At the end of the experiment, JL-PRL mice exhibited lower serum ALT, AST, and TG levels, as well as decreased hepatic fat deposition, as revealed by MRI, Oil Red O staining, H&E staining, and electron microscopy compared with the JL-vehicle group, and this effect was more pronounced when PRL was administrated at ZT12 (Fig. 6d–f). mRNA levels of lipogenic genes were detected and the results showed that decreased *Ccnd1* mRNA levels in the liver of jetlag mice were up-regulated after PRL treatment, and a more obvious up-regulation was seen in JL-PRL12 compared to JL-PRL0. In addition, elevated expressions of *Fasn*, *Acc,* and *Cidea* in the liver of jetlag mice were markedly reversed in the JL-PRL group, in which PRL treatment at ZT12 decreased hepatic *Acc*, *Fasn*, and *Cidea* expressions to a greater extent compared with PRL treatment at ZT0. However, *Pparγ* levels were not significantly altered after PRL treatment (Fig. 6g, Additional file 1: Fig. S9e). Western blotting analysis also confirmed that down-regulated levels of CCND1, and up-regulated FASN and ACC protein levels in the liver of jetlag mice were reversed after PRL treatment, particularly at ZT12 (Fig. 6h).

## Discussion

**Fig. 7:**
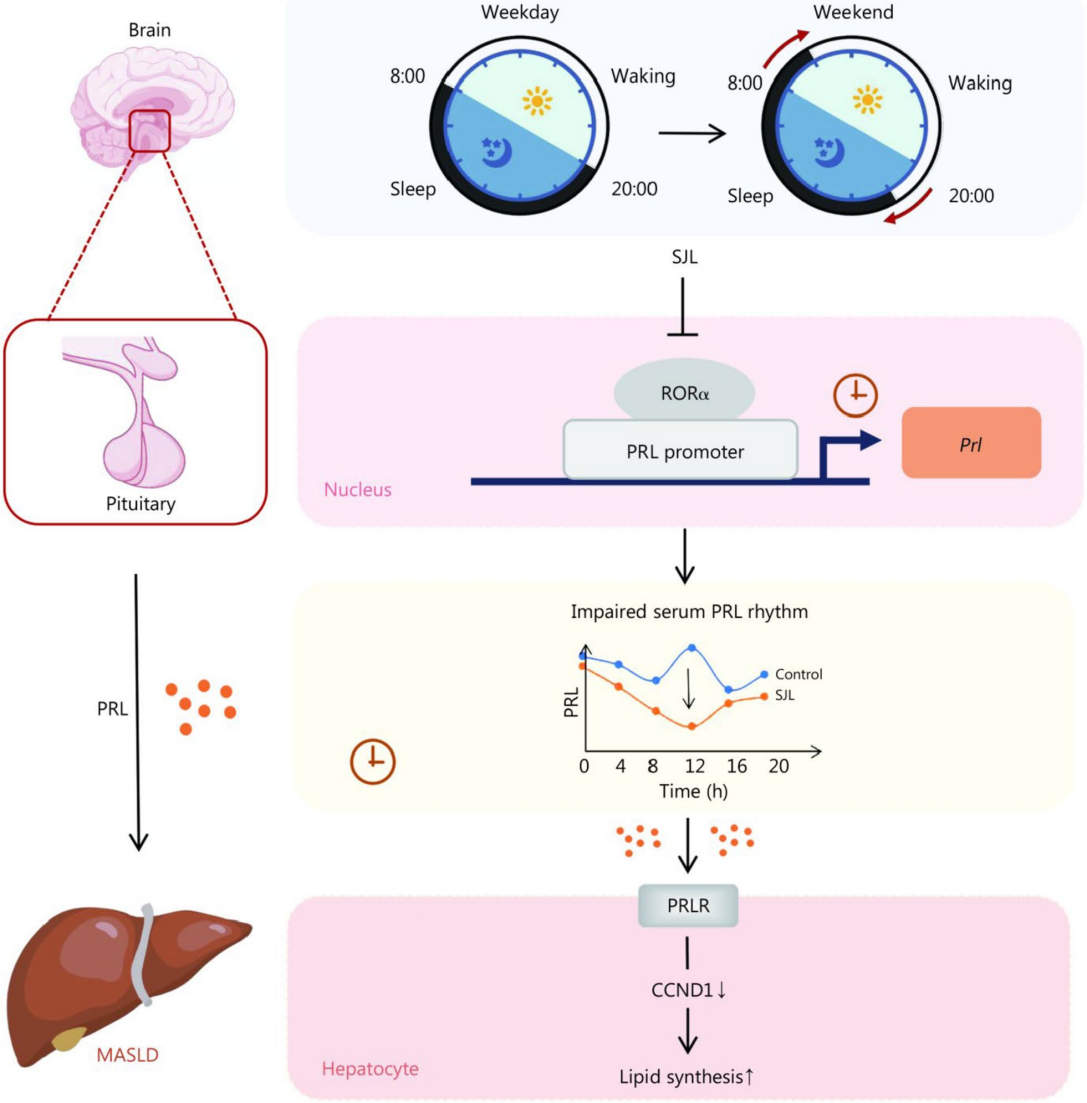
Working model of this study. Social jetlag (SJL) induces MASLD via SJL-driven flattened serum prolactin (PRL) rhythm, in which SJL rewires the hepatic circadian transcriptome of the PRL signaling pathway. Consequently, suppressed hepatic mitogen-activated protein kinase (MAPK)/cyclin D1 (CCND1) signaling mediated lipogenesis contributes to SJL-induced fatty liver. MASLD metabolic dysfunction-associated steatotic liver disease, RORα retinoic acid receptor-related orphan receptor α

In this study, we first identified SJL as a risk factor for MASLD, which is mediated through a disrupted PRL rhythm due to compromised transcription by RORα. Mechanistically, SJL reprogrammed the hepatic circadian transcriptome of the PRL signaling pathway, and then up-regulating lipid synthetic genes in the liver (Fig. 7). Furthermore, our findings suggested that the timing of drug therapy should be considered an important factor in optimizing MASLD treatment.

SJL induces circadian misalignment that may dampen neural and hormonal signals’ outflow from central to peripheral organs. In this study, blood pressure levels, representing proxy measurements of the autonomic nervous system, were not significantly different between subjects with and without SJL, suggesting that SJL had a marginal effect on the neural system. Thus, our focus shifted to alterations in hormonal regulation under SJL conditions, and it was found that only pituitary-derived PRL was affected by SJL. PRL, historically known for its role in stimulating mammary glands and lactogenesis, is a pleiotropic polypeptide with receptors widely distributed across various metabolic organs in both males and females [19]. Moreover, unlike other adenohypophyseal hormones such as TSH, FSH, LH, and ACTH, which are mainly regulated by classical negative feedback, PRL lacks a specific target endocrine gland and is more likely to respond to environmental changes. Indeed, the characteristic nocturnal rise of PRL is disrupted under circadian misalignment environments like night light exposure and night eating syndrome [20, 21]. In our study, the rhythm of PRL was perturbed in subjects with SJL which mediated the pathogenic effect of SJL on MASLD, revealing PRL as the most important pituitary hormone that is affected by environmental factors.

Murine studies often implement changes in the light–dark (LD) cycle to simulate SJL in humans [22–24]. According to previous literature, the effect of an 8-h phase light delay in mice on the rhythm of activity movement was too large and the mice did not return to normal after more than 2 weeks [25]. Another study with 3-h delay for 2 d following 5 d of the normal LD cycle reported that the range of movement was so subtle that the activity rhythm was not affected [26]. Therefore, the model used here was a 2-h LD delay every 3 days to mimic SJL in humans. Consistent with previous findings in jetlagged mice, we observed that 16 weeks of jetlag resulted in increased liver injury markers (ALT, AST), as well as elevated TG levels and hepatic steatosis. Similar to clinical data, SJL altered PRL rhythmicity in mice at ZT12. Importantly, the inhibition of PRL by bromocriptine or by knocking out *Prl* in vivo under JL showed a similar degree of liver steatosis compared with their counterparts. These findings suggest that SJL-induced fatty liver is mediated by disturbed PRL rhythm.

The mammalian circadian clock, comprises a transcription feedback network involving clock genes *Clock*, *Bmal1*, and nuclear receptors RORs and REVERBs [27] which are expressed in a circadian manner in vivo [28]. Analysis of the PRL proximal promoter revealed the presence of an RORE (5’-AGGTCA-3’), and it served as a binding site of transcription factor RORα and REVERBα, which mediated the activation of target genes. Although it is well established that RORα and REVERBα were oscillated in vivo, studies regarding the rhythm of these nuclear transcription factors in the pituitary were rare. We first investigated the circadian characteristics of RORα in mice pituitary and showed that the peak value of RORα expression was around ZT12. This study also adds to the evidence that the RORE of the *Prl* promoter could bind RORα and thus transactivate *Prl* expression, yet this effect was dampened by SJL.

How does disrupted PRL rhythm participate in the process of SJL-related MASLD? Transcriptomic changes in liver tissue from subjects with SJL and MASLD revealed enrichment of DEGs in the PRL signaling pathway compared with their respective controls. More importantly, we reconstructed the human hepatic circadian transcriptome atlas using the ZeitZeiger algorithm on RNA-seq data from 197 human liver tissues. ZeitZeiger is a novel and state-of-the-art supervised machine learning algorithm for analyzing circadian information of RNA-seq data and the core idea of this algorithm is dimensionality reduction [29]. It consists of finding a suitable low-dimensional manifold and then identifying the one-dimensional trajectory (circadian time) for the testing set that best matches the known time-labeled samples from the training set. The algorithm’s prediction accuracy was assessed using MAE, which was calculated as the mean of the differences between the observed and predicted times of each sample. We used this metric to represent the deviation of circadian rhythm in our model of gene orchestration. In the present study, subjects with SJL < 1 h and ≥ 1 h were used as the training datasets and testing datasets, respectively. Higher MAE observed in the SJL group indicated that differences between predicted and observed times were larger in SJL subjects than in controls. Importantly, circadian curves of observed times in the PRL signaling pathway, circadian rhythm, and NAFLD deviated from predicted times in SJL subjects, suggesting an alteration of circadian expressions of genes related to these pathways in the liver under SJL conditions.

Hepatic steatosis is the result of an imbalance between lipid synthesis and utilization [30]. Our validation results showed that only CCND1 was significantly down-regulated in the liver of SJL mice at ZT12. CCND1, a key cell cycle protein, inhibits de novo lipogenesis in hepatocytes by diminishing FASN and ACC expressions [18]. Previous studies indicated that CCND1 was transactivated by STAT proteins, MAPKs, and phosphatidylinositol 3 kinase (PI3K) [31–33], which are canonical downstream effectors of PRL signaling pathways [34]. These findings suggest that CCND1 might play a crucial role in SJL-related fatty liver. Indeed, PRL significantly up-regulated CCND1 and diminished FASN and ACC expression in HepG2 cells. Importantly, SB203580 (MAPK inhibitor) not only abrogated the PRL-stimulated CCND1 expressions but also abolished the inhibitory effect of PRL on FASN and ACC expressions, while LY294002 (PI3K/Akt inhibitor) and STAT5-IN-1 (STAT5 inhibitor) did not show this effect. Physiologically, CCND1, FASN, and ACC were rhythmically expressed in the liver, integrating lipid metabolism [35, 36], we adopted serum shock to synchronize the rhythms of cyclic genes to mimic circadian conditions in vitro. Serum shock analysis demonstrated that the lack of *Prl* led to a loss of rhythmicity in *Ccnd1*, *Fasn*, and *Acc*, indicating that alterations in lipogenic genes were a consequence of dysregulated PRL signaling. Based on these data, decreased serum PRL levels at ZT12 under SJL might inhibit hepatic CCND1 levels through MAPKs, leading to the activation of lipogenic enzymes (i.e., FASN and ACC) and hepatic steatosis. Furthermore, clock genes including hepatic *Per3*, *Reverbα*, and *Rorc* were significantly altered in the SJL group at ZT12, suggesting that circadian rhythm may also participate in SJL-induced hepatic steatosis.

Chronotherapy refers to administering drugs at times when they are most effective. In the liver, the expression of genes critical for de novo lipogenesis has rhythmic expression patterns, thus time of drug administration might be integrated to acquire more favorable therapeutic outcomes in treating MASLD [37]. In the liver, FASN, and ACC involved in lipid synthesis exhibit circadian fluctuations, which are relatively more highly expressed at night than those during the day [38]. Here, we observed that PRL treatment at ZT12 under SJL conditions improved lipid metabolism to a greater extent than that at ZT0. Therefore, the maximized therapeutic effect of PRL supplementation at ZT12 might be attributed to increased FASN and ACC expression at this time. These results support the notion that restoring the circadian rhythm of the central nervous system might enhance the effectiveness in treating MASLD, in particular, PRL might serve as the chronotherapeutic target in this disease.

There were several limitations of the present study: First, PRL levels were detected only at 3-time points in a clinical setting, which might not cover the overall circadian characteristics of PRL. However, a previous study [39] that sampled subjects’ blood every 3 h showed that peak PRL levels occurred between 23:00 and 02:00, and remained at relatively higher levels until 8:00, and then decreased through daytime, especially around 14:00. Therefore, the 3 time points (8:00, 16:00 and 24:00) of PRL sampling we chose in our study could better represent serum PRL rhythm during 24 h. Second, human liver biopsy samples that were analyzed for RNA-seq were obtained during 8:00–19:00; therefore, ZeitZeiger analysis may not reflect circadian features of the liver throughout the whole day. However, we have investigated the rhythm of a hepatic transcriptome based on strictly 4-h intervals during 24 h in murine models, obtaining similar findings. Therefore, we concluded that the hepatic PRL signaling pathway, circadian rhythm, and lipid metabolism were dys-synchronized under SJL. The third limitation is that we only used female mice to examine the mechanism of jetlag-induced fatty liver. However, a previous study has shown that PRL intervention could also reduce hepatic steatosis in male mice under HFD [40]. In addition, we have adjusted sex in regression analysis regarding SJL-associated MASLD in clinical cohort and the conclusions remained consistent. Therefore, our findings exist in both males and females. Lastly, the subjects included in the clinical study incorporate those who are prone to receive liver biopsy, and a larger sample size of population-based study needs to be carried out in the future to corroborate this conclusion.

Collectively, our study establishes SJL as a risk factor for histologically proven MASLD, and disturbed PRL rhythm participates in this process. Disrupted PRL rhythm caused by impaired transcriptional regulation of RORα in the pituitary, accompanied by disturbed oscillation of PRL signaling pathway and the resulting heightened lipogenesis in the liver, contributed to SJL-induced fatty liver. Finally, restoring circulating PRL rhythm may be a new strategy in treating SJL-associated MASLD.

## Conclusions

In the present study, we demonstrated for the first time that SJL induces fatty liver via flattened serum PRL rhythm, in which the transcriptional activation of pituitary PRL by RORα was dampened under jetlag. In this regard, disrupted PRL rhythm rewired hepatic circadian transcriptome of PRL signaling pathway and consequently up-regulated hepatic lipogenesis via suppressed hepatic MAPK/CCND1 pathway. Importantly, restoration of PRL rhythm could alleviate SJL induced fatty liver more effectively compared with conventional PRL administration. Our work emphasized that PRL played an important role in mediating SJL related fatty liver and that the timing of drug therapy should be taken into consideration in optimizing MASLD treatment.

## Supplementary Information

**Additional file 1. Methods. Table S1** Baseline characteristics of the study population categorized by social jetlag levels. **Table S2** SJL profiles of the study population with and without MASLD [*n* (%)]. **Table S3** Primers used for qRT-PCR assays. **Fig. S1** Illustration of the flowchart of the study population (a) and the correlation between jetlag and pituitary hormones and steatosis severity (b). **Fig. S2** The effect of social jetlag (SJL) on energy homeostasis. **Fig. S3** Overexpression and knockdown of Reverbα and Rorα in vitro. **Fig. S4** Kyoto Encyclopedia of Genes and Genomes (KEGG) enrichment pathways based on up- and down-regulated DEGs from SJL vs. control subjects. **Fig. S5** Kyoto Encyclopedia of Genes and Genomes (KEGG) enrichment pathways of circadian genes in the liver of female mice under normal light cycle (NC). **Fig. S6** Kyoto Encyclopedia of Genes and Genomes (KEGG) enrichment of circadian genes using microarray data in male mice under normal light cycle retrieved from public Gene Expression Omnibus (GEO) database). **Fig. S7** Kyoto Encyclopedia of Genes and Genomes (KEGG) enrichment pathways of circadian genes in the liver of female mice under jetlag (JL). **Fig. S8** Jetlag (JL) altered hepatic transcriptome of mice at ZT12. **Fig. S9** Expression of differentially expressed genes between normal light cycle (NC) and jetlag (JL) group at ZT12 and the effect of prolactin (PRL) treatment.

## Data Availability

The data that support the findings of this study are available from the corresponding author upon reasonable request.

## Abbreviations

ACTH: Adrenocorticotropic hormone
ACC: Acetyl-CoA carboxylase
Akt: Protein kinase B
ALT: Alanine aminotransferase
AST: Aspartate aminotransferase
AUC: Area under the curve
CCND1: Cyclin D1
BMI: Body mass index
DEG: Differential expression gene
FASN: Fatty acid synthase
FBG: Fasting blood glucose
FSH: Follicle-stimulating hormone
GH: Growth hormone
GTT: Glucose tolerance test
Gucy1a1: Guanylate cyclase 1 soluble alpha 1
Ndufs5: NADH ubiquinone oxidoreductase subunit S5
HbA1c: Hemoglobin A1c
HDL: High-density lipoprotein cholesterol
HOMA-IR: Homeostatic model assessment for insulin resistance
ITT: Insulin tolerance test
JL: Jetlag
KEGG: Kyoto encyclopedia of genes and genomes
LD: Light–dark
LDL: Low-density lipoprotein cholesterol
LH: Luteinizing hormone
MAE: Mean absolute error
MAPK: Mitogen-activated protein kinase
MASLD: Metabolic dysfunction-associated steatotic liver disease
MRI: Magnetic resonance imaging
mTOR: Mechanistic target of rapamycin kinase
NAFLD: Non-alcoholic fatty liver disease
NC: Normal light cycle
PCA: Principal component analysis
PI3K: Phosphatidylinositol 3-kinase
PRL: Prolactin
Pparγ: Peroxisome proliferator-activated receptor γ
PPI: Protein–protein interaction
REVERBα: Nuclear receptor subfamily 1 group D member 1
RNA-seq: RNA-sequencing
RORα: Retinoic acid receptor-related orphan receptor a
SJL: Social jetlag
SPC: Sparse principal component
STAT: Signal transducer and activator of transcription
TC: Total cholesterol
TG: Triglyceride
TSH: Thyrotropin-stimulating hormone
