# Supplementary material for: Social jetlag elicits fatty liver via perturbed circulating prolactin rhythm-mediated circadian remodeling of hepatic lipid metabolism
Source: Mil Med Res. 2025 Jun 3;12:29. doi: 10.1186/s40779-025-00609-z (PMC12131380; doi:10.1186/s40779-025-00609-z)
Supplement: Supplementary file 1 — Additional file 1. Methods. Table S1 Baseline characteristics of the study population categorized by social jetlag levels. Table S2 SJL profiles of the study population with and without MASLD [n (%)]. Table S3 Primers used for qRT-PCR assays. Fig. S1 Illustration of the flowchart of the study population (a) and the correlation between jetlag and pituitary hormones and steatosis severity (b). Fig. S2 The effect of social jetlag (SJL) on energy homeostasis. Fig. S3 Overexpression and knockdown of Reverbα and Rorα in vitro. Fig. S4 Kyoto Encyclopedia of Genes and Genomes (KEGG) enrichment pathways based on up- and down-regulated DEGs from SJL vs. control subjects. Fig. S5 Kyoto Encyclopedia of Genes and Genomes (KEGG) enrichment pathways of circadian genes in the liver of female mice under normal light cycle (NC). Fig. S6 Kyoto Encyclopedia of Genes and Genomes (KEGG) enrichment of circadian genes using microarray data in male mice under normal light cycle retrieved from public Gene Expression Omnibus (GEO) database). Fig. S7 Kyoto Encyclopedia of Genes and Genomes (KEGG) enrichment pathways of circadian genes in the liver of female mice under jetlag (JL). Fig. S8 Jetlag (JL) altered hepatic transcriptome of mice at ZT12. Fig. S9 Expression of differentially expressed genes between normal light cycle (NC) and jetlag (JL) group at ZT12 and the effect of prolactin (PRL) treatment. [file 40779_2025_609_MOESM1_ESM.docx]

**Methods**

**Clinical cohorts**

***Participants recruitment***

Eligible participants aged between 18 and 57 years who received a liver biopsy according to the routine clinical practice to confirm the diagnosis of fatty liver disease were recruited from Nanjing Drum Tower Hospital between January 2019 and November 2021. The time information of the liver biopsy was recorded for each subject. Biopsy specimens were evaluated by two expert pathologists. Metabolic dysfunction-associated steatotic liver disease (MASLD) was defined by > 5% macrovesicular steatosis, together with at least 1 out of 5 cardiometabolic criteria, e.g., presence of impaired glucose regulation, type 2 diabetes, overweight or obesity, hypertension, or dyslipidemia [1]. The sleep information was recorded for each participant using an adapted version of the Munich ChronoType Questionnaire [2]. Social jetlag (SJL) was calculated as the absolute difference between midsleep time points on workdays and free days and further dichotomized as < 1 h or ≥ 1 h. Participants were categorized into control (SJL < 1 h, *n* = 125) and SJL (SJL ≥ 1 h, *n* = 72) groups based on the disparity between the midpoint of weekday and weekend sleep. If a subject experienced negative SJL values, absolute values were used for calculation. The following conditions were excluded from the study: diseases or medication usage that may affect prolactin (PRL) levels (pituitary adenoma, corticosteroids, and antipsychotic drugs), known hyperthyroidism or hypothyroidism, malignant tumor, type 1 diabetes, pregnant or postpartum females in breastfeeding. Anthropometric variables, including body mass index (BMI), waist and hip circumference, and systolic and diastolic blood pressure were measured according to standard procedures. Circulating concentrations of alanine aminotransferase (ALT), aspartate aminotransferase (AST), triglyceride (TG), total cholesterol (TC), low-density lipoprotein cholesterol (LDL-C), and high-density lipoprotein cholesterol (HDL-C) were determined through an autoanalyzer (Abbott Laboratories, USA). Hemoglobin A1c (HbA1c) was measured by high-performance liquid chromatography (Bio-Rad D-10, Japan). Fasting blood glucose (FBG) levels were examined using a hexokinase method (TBA-200FR, Japan). Fasting insulin (FINS) was quantified by electrochemiluminescent immunoassay (Roche, USA). Homeostasis model assessment of insulin resistance (HOMA-IR) = FBG (mmol/L) × FINS (μIU/L)/22.5 [3]. The protocol of the present study conformed to the guidelines of the Declaration of Helsinki and was approved by the Ethics Committee of Nanjing Drum Tower Hospital, Nanjing University Medical School (2021-388-01). All participants signed informed consent before study inclusion.

***Sample size estimation***

The sample size herein was calculated based on the formula adapted to the case-control study using PASS software (the detail was in the following figure): the threshold of α was set at 0.05 and β at 0.15, Zα was 1.96. According to previous epidemiological studies [4, 5], the rate of exposure to SJL in the control group (p0) was estimated to be 45%. Another recent study demonstrated that the odds ratio of circadian misalignment such as disturbance in nighttime sleep on MASLD was 2.38, (95% CI 1.73-3.27) [6]. In the sample size calculation, the odds ratio of SJL on MASLD was estimated to be 2.5.

Sample size in the mouse studies was chosen based on previous literature for experimental procedure [7]. The sample size of mice in JTK_CYCLE analysis is three per group at each time point, according to previous studies [8].

***Circadian analysis of pituitary hormones***

A blood sample was taken from the cubital vein of each participant for pituitary hormone detection at 8:00, 16:00, and 24:00 on the same day. Serum PRL, adrenocorticotropic hormone (ACTH), growth hormone (GH), follicle-stimulating hormone (FSH), luteinizing hormone (LH), and thyrotropin-stimulating hormone (TSH) levels were quantified using an automated chemiluminescent immunoassay (Siemens Immulite 2000, Germany). Employing a 3-time point rhythm prediction method grounded in multiple linear regression, sine-wave models for each subject were fitted to these hormone profiles, enabling the determination of amplitude and phase values: *F(t)* = a + b × sin(2π × $\frac{t}{24}$) + c × cos(2π × $\frac{t}{24}$), and amplitude = (b^2^ × c^2^)^1/2^, phase = arctan (c/b) [9].

**In vivo study**

C57BL/6 female mice (*n* = 130) were procured from Gempharmatech Co., Ltd., China (license No. A202411270106). All animal studies, including the euthanasia procedure for the mice, were conducted in adherence to the regulations and guidelines of the Animal Ethics Committee of Nanjing Drum Tower Hospital (2021AE01044), following institutional animal care standards and following the guidelines of the Association for Assessment and Accreditation of Laboratory Animal Care International (AAALAC) and Institutional Animal Care and Use Committee (IACUC).

***Jetlag model***

Eight-week-old C57BL/6J female mice were housed in either a normal 12 h:12 h light/dark (LD) cycle or subjected to an SJL condition. In the SJL schedule, mice experienced normal-LD conditions for 3 d followed by 2-hour delayed-LD conditions for the subsequent 3 d each week. The normal LD condition was defined as 8:00 am to 8:00 pm, while zeitgeber time (ZT) 0 and ZT12 were defined as the time when the lights were on and off, respectively. In the jetlag (JL) group, ZT times were synchronized (i.e., ZT0 of JL mice corresponded to ZT0 of control mice). Throughout the study, all mice had unrestricted access to food and water. After 16 weeks, serum was collected using the retro-orbital technique at 4-hour intervals corresponding to ZT: ZT0, ZT4, ZT8, ZT12, ZT16, and ZT20. All samples were taken on the same day after mice in the JL group experienced 37 jetlag shifts. After 16 weeks of jetlag, all mice (24 weeks of age) were culled starting at ZT0 (8:00 am) in mice under normal light cycle and ZT0 (8:00 am) in JL mice. For blood draws, all blood measures were collected consecutively at a 4-hour interval during the 24-hour course. When samples were collected in the dark phase, we used the dim light during the euthanasia of mice and sample collection to minimize the influence of light exposure on gene expressions. Serum samples from these 6 time points were processed for the detection of circulating PRL.

***Bromocriptine intervention***

Eight-week-old C57BL/6 female mice were maintained under a 12 h:12 h LD cycle. Bromocriptine mesylate (5 mg/kg, Aladdin, China) was intraperitoneally injected daily at ZT12 for 10 weeks. Lipid content was evaluated every 2 weeks by magnetic resonance imaging (MRI) scanning.

***Prl depletion***

*Prl*-knockout (*Prl*^-/-^) female mice (*n* = 15) were generated using CRISPR-Cas9 technology on the C57BL6/J background in our lab [10]. At 8 weeks of age, these *Prl*^-/-^ female mice were allocated to either a normal or jetlag-mimicking LD cycle, following the protocol described above, and were maintained in their respective conditions for 16 weeks.

***PRL intervention***

Wild-type mice, under both normal light cycles and jetlag conditions, received intraperitoneal injections of either PBS or PRL (Novoprotein Corp., China) at a dosage of 0.025 mg/d at ZT12 daily for 2 weeks.

***Glucose tolerance test (GTT)/insulin tolerance test (ITT)***

For the GTT, mice were fasted for 8 h and were injected i.p. with glucose (1.5 g/kg body weight). For the ITT, mice were fasted for 4 h and were injected i.p. with insulin (0.75 U/kg body weight). Tail vein blood was collected, and blood glucose levels at each time point were measured using Accu-Chek Performa (Roche, Germany).

***Hormones detection***

PRL levels in mice serum at ZT0, ZT4, ZT8, ZT12, ZT16, and ZT20, and supernatant of rat MMQ cell were detected by the mouse (FcMACS, China) and rat enzyme-linked immunosorbent assay (ELISA) kit (Raybiotech, USA), respectively.

***Liver MRI scanning***

In vivo, imaging was acquired on a horizontal 20 cm-bore 9.4 T MRI animal scanner (Biospec 94/20USR, Bruker, Germany). After anesthesia with isoflurane, mice were placed in plastic cradles in the supine position and slid into the imaging coil. Axial T1-weighted fat-water separation imaging was performed with the following parameters: average = 2, echo times = 7 ms, repetition time = 600 ms. The region of interest (ROI) was positioned on the right side close to the stomach to avoid motion artifact of the lung. From the separated fat (Sf) and water (Sw) signals, a fat-fraction signal (η) of the ROI was calculated as: η = Sf/(Sf + Sw) for each voxel, and the mean η over the 3 voxels was used in the data analysis.

***Liver function and histological evaluations***

Serum ALT, AST, and TG contents were evaluated using the ELISA kit (Jiyinmei, Wuhan, China). Photometric absorbance was read at 450 nm.

For Oil Red O staining, frozen liver tissues of mice were embedded in the OCT compounds and sliced into sections at 5 µm and fixed with 4% formaldehyde, after which the sections were stained in 0.5% Oil Red O solution prepared for hematoxylin counterstaining. For H&E staining, liver tissues of mice were fixed overnight with 4% paraformaldehyde, embedded in paraffin, and cut into slices at 5 µm. Slides were washed and incubated with eosin for 30 min. Images were acquired through a Panoramic slide scanner (3DHistech Ltd., Hungary).

For transmission electron microscopy, 1 mm^3^ livers were fixed with 2.5% glutaraldehyde and postfixed with 1% osmium tetroxide. After being dehydrated in graded ethanol, samples were embedded in EMBed 812 (SPI, USA) and polymerized under 65 ℃ for 48 h. 60 – 80 nm sections were stained with uranium acetate and lead citrate and were examined under a transmission electron microscope (HT7800, Hitachi, Japan).

**Cell experiments**

***Cell culture***

PRL-secreting rat pituitary cell line (MMQ cells) and 293T cells were obtained from the Cell Bank of the Chinese Academy of Sciences. The MMQ cells were cultured in Ham’s F12 medium with 10% fetal bovine serum (FBS) and 1% penicillin-streptomycin. The growth media of 293T cells is DMEM with 10%FBS and 1% penicillin-streptomycin. Primary hepatocytes were isolated from female wild-type C57BL/6 or *Prl^-/-^* mice. Briefly, the mice’s liver was perfused with Hank’s solution and digested with a buffer containing 0.04% collagenase (Type IV, Sigma). The cell suspensions were filtered through a 100 μm cell strainer and centrifuged to remove non-hepatocyte cells. Primary hepatocytes were then cultured in William’s E medium supplemented with 10% FBS and 1% penicillin-streptomycin.

HepG2 cells (obtained from Cell Bank of the Chinese Academy of Sciences), after a 12-hour incubation in serum-free DMEM, were treated with specific inhibitors at designated concentrations [mitogen-activated protein kinase (MAPK) inhibitor (SB203580, 10 µmol/L, MCE, China), phosphoinositide 3-kinases-serine/threonine kinase 1 (PI3K/Akt) inhibitor (LY294002, 20 µmol/L, MCE, China), or signal transducer and activator of transcription 5 (STAT5) inhibitor (STAT5-IN-1, 50 µmol/L, MCE, China)] 2 h before PRL intervention (24 h, 100 µg/L) (Novoprotein Corp., China).

***Lentiviral preparation and cell transfection***

The full-length coding sequence of the rat retinoic acid receptor-related orphan receptor α (*RORα*; Gene ID: 6095) and nuclear receptor subfamily 1 group D member 1 (*Reverbα*; Gene ID: 252917) was cloned into pHBLV (CMV-MCS-3flag-EF promoter-zsGreen1-T2A-Puro). Subsequently, it was co-transfected with pSPAX and pMD2.G into 293T cells. The purified products were added to infect MMQ cells.

Lentivirus short hairpin RNA (shRNA) against RORα (5’-GGATCAAACCCGAACCCATAT-3’), Reverbα (5’-CAGCAACATTACCAAGCTTAA-3’), and nonspecific vehicle (5’-TTCTCCGAACGTGTCACGT-3’) were constructed using the GV644 vector (Genechem Co., Ltd., China). Lentivirus was transfected into MMQ cells at 60 to 80% confluence. Cells were collected for mRNA and ELISA detection after 72 h of transfection.

The expressions of *RORα* and *Reverbα* were detected using qRT-PCR.

***Serum shock***

For serum shock experiments, primary hepatocytes from wild or *Prl^-/-^* mice were treated with 50% horse serum (Gibco, USA). After a 2-hour serum shock synchronization, cells were washed and given serum-free DMEM, with the initial time recorded as ZT0. Cells were collected every 4 h after the serum shock experiment, lasting for 24 h (ZT0, ZT4, ZT8, ZT12, ZT16, and ZT20), and RNA was extracted at each time point for qRT-PCR analysis. The rhythmicity of gene expression in primary hepatocytes from mice was tested by the CircaCompare algorithm (https://rwparsons.shinyapps.io/circacompare/).

**mRNA analysis**

Total RNA from MMQ and 293T cells, primary hepatocytes, and liver tissues were extracted by Trizol reagent (Invitrogen, USA) and reverse-transcribed using the Reverse Transcription Kit (TaKaRa, Japan). Quantitative real-time PCR was analyzed by SYBR Green on Light Cycler 480 System (Roche, Switzerland). The used primers are listed in the **Additional file 1: Table S3**.

**RNA sequencing**

***Human liver specimen***

Strand-specific paired-end libraries were generated from total RNA using NEBNext^®^ Ultra™ RNA Library Prep Kit and run on an Illumina NovaSeq 6000 sequencer to obtain 150 bp end reads. Low-quality reads were removed, and the filtered reads were mapped to the GRCh38 human reference genome. The featureCounts v1.5.0-p3 tool was employed to count the read numbers mapped to each gene. Differential expression gene (DEG) analysis was performed using the DESeq2 R package, with a log_2_ fold change (FC) cutoff threshold of 1.2 and an adjusted *P*-value < 0.01. DAVID was employed for functional annotation clustering of DEGs, with an enrichment threshold of *P* < 0.05.

The ZeitZeiger algorithm was applied to infer the circadian features of human liver tissue based on the sample timing (observed time) of the liver from each subject and gene abundance from RNA-seq data [11]. Two main parameters, sumabsv and sparse principal component (SPC), were involved. Sumabsv controls how many genes form each, and the number of SPCs determines how many SPCs are used for prediction (predicted time), with the number of SPCs being optimized by cross-validation.

Hepatic gene expression of subjects in the control group (training datasets) was used to fit ZeitZeiger, and predictions were generated on the SJL subjects (testing datasets) for comparison. The performance of the prediction was evaluated by the mean absolute error (MAE) between the observed time and the predicted time, where around 1 h signifies an accurate performance [11]. The circadian features of gene expression in SJL subjects were evaluated by comparing the MAE difference between predicted (predicted time) and known sample timing information (observed time).

***Mice liver tissue***

Strand-specific paired-end libraries were generated from total RNA using NEBNext^®^ Ultra™ RNA Library Prep Kit and run on an Illumina NovaSeq 6000 sequencer to obtain 150 bp end reads. To ensure data quality, low-quality reads were removed, and the filtered reads were mapped to the mouse genome GRCm39 (https://[www.ncbi.nlm.nih.gov/assembly/GCF_000001635.27).](http://www.ncbi.nlm.nih.gov/assembly/GCF_000001635.27)) The featureCounts v1.5.0-p3 tool was employed to count the read numbers mapped to each gene. DEG analysis was performed using the DESeq2 R package (version 1.20.0), the cutoff threshold of log_2_ FC was set at 1, and the adjusted *P*-value was maintained at < 0.05. Time-series expression profiles were subjected to the nonparametric test Jonckheere-Terpstra-Kendall (JTK_CYCLE), version 3, to analyze periodicity [12]. A window of 20 – 28 h was utilized for determining periodicity, and statistical significance was defined by *P* < 0.05. Functional pathway analysis of cyclic genes was carried out using the DAVID database online. Additionally, network analysis was performed using the Search Tool for the Retrieval of Interacting Genes (STRING) version 11.5.

In addition, microarray data from the Gene Expression Omnibus (GEO) profiles public database (accession No. GSE52333) [13] were incorporated into the analysis. This dataset involved microarray data from the liver of C57BL/6J male mice maintained in normal light conditions and the rhythmicity was tested via JTK_CYCLE.

**Transcriptional analysis**

Potential transcription factor binding sites of PRL were analyzed on the 2000 bp upstream segment of homo sapiens, rattus norvegicus, and mus musculus PRL, utilizing the JASPAR database ([http://jaspar.genereg.net/).](http://jaspar.genereg.net/))

For the luciferase reporter assay, 293T cells (obtained from the Cell Bank of the Chinese Academy of Sciences) were cultured until reaching 70% confluency. Transient transfection was performed using Lipofectamine 2000 (Invitrogen, USA), incorporating 2 µg of the luciferase reporter plasmid, 2 µg of the expression construct, and 200 ng of the Renilla luciferase plasmid. Following a 48-hour incubation period, luciferase assays were executed employing a dual-luciferase assay kit (Promega, Madison, USA). The obtained results were subsequently normalized by Renilla luciferase activity.

For the analysis of chromatin immunoprecipitation, approximately 150 mg of frozen pituitary tissue from mice was finely minced and fixed with 37% formaldehyde. The cross-linking process was arrested by the addition of 0.125 mol/L glycine, facilitating the cross-linking of DNA and chromatin-binding proteins. Subsequently, the cross-linked samples were sonicated into fragments ranging from 200 to 600 bp and left to incubate overnight with the specified antibodies. ChIP-grade protein A/G agarose beads (9007S, Cell Signaling Technology, USA) were then introduced and allowed to incubate for 2 h. The DNA-antibody-bead complex underwent reversal at 65 °C overnight in the elution buffer and was subjected to digestion with RNase A and proteinase K. Following this, the DNA was purified using an organic solvent, and the enrichment of ChIP DNA was analyzed through qRT-PCR.

The electrophoretic mobility shift assay (EMSA) analysis was conducted on human nonfunctioning pituitary tissue, diagnosed based on normal hormone levels and negative immunohistochemical staining. Nuclear extracts were derived from pituitary tissue using NE-PER nuclear and cytoplasmic extraction reagents (Pierce, IL, USA). Biotin-labeled oligonucleotides and mutated oligonucleotides were as follows: 5’-CAAATTTGAAACTAAAGGTCACAGGCTGCTTTAGA-3’ and 5’-CAAATTTGAAACTAAGAACTGCAGGCTGCTTTAGAT-3’, respectively. EMSA was conducted using a commercial kit (ThermoFisher Scientific, IL, USA). The protein-DNA complexes were separated on a 5% polyacrylamide non-denaturing gel and subsequently transferred to a nylon membrane (Pierce, IL, USA). Visualization of the probes was accomplished using the ECL reagent.

**Western blotting**

293T cells were transfected with plasmids overexpressed *Reverba* and *Rora* and were lysed in IP buffer. After centrifugation (12,000 rpm, 15 min, 4 °C), the supernatants were incubated overnight at 4 °C. The precipitates were washed and subjected to SDS-PAGE and immunoblotting analysis with anti-Flag and anti-GAPDH antibodies (CST, Danvers, MA, USA). HepG2 cells were lysed using RIPA buffer, and total protein was extracted for Western blotting analysis. The primary antibodies employed in this analysis included β-actin (CST, USA), fatty acid synthase (Fasn), acetyl coenzyme A carboxylasel (Acc), and cyclin D1 (Ccnd1) (Proteintech, China).

**Statistical analysis**

Shapiro-Wilk normality test was performed to assess the normality of the data. For the clinical study, data were expressed with mean ± standard deviation (SD) if they were normally distributed, and were tested by Students’ *t*-test. Skewed distribution data were presented as *M* (*Q*_1_, *Q*_3_) and were analyzed by the Mann-Whitney *U* test. Categorical variables were expressed as *n* (%) and were analyzed using the *χ*^2^ test. Logistic regression and mediation analysis were used to estimate the association between SJL, PRL rhythm, and MASLD. Principal-component analysis (PCA) was used to determine the parameters associated with MASLD. Correlation analysis, PCA analysis, and mediation analysis were performed in R software (4.1.2). For mice and cell experiments, data were presented as scatter dot plots and were analyzed by Students’ *t*-test or one-way ANOVA with Tukey’s post hoc tests. SPSS 22.0 (SPSS 22.0 Inc., USA) was used for statistical analysis. Figures were created in GraphPad Prism software (version 9) and R software (version 4.2.1). *P* < 0.05 indicated statistical significance (two-tailed).

**References**

1 Rinella ME, Lazarus JV, Ratziu V, Francque SM, Sanyal AJ, Kanwal F, et al. A multisociety Delphi consensus statement on new fatty liver disease nomenclature. J Hepatol. 2023;79(6):1542-56.

2 Kantermann T, Sung H, Burgess HJ. Comparing the Morningness-Eveningness Questionnaire and Munich ChronoType Questionnaire to the Dim light melatonin onset. J Biol Rhythms. 2015;30(5):449-53.

3 Petrelli A, Cugnata F, Carnovale D, Bosi E, Libman IM, Piemonti L, et al. HOMA-IR and the Matsuda Index as predictors of progression to type 1 diabetes in autoantibody-positive relatives. Diabetologia. 2023;67(2):290-300.

4 Koopman ADM, Rauh SP, Van ‘T Riet E, Groeneveld L, Van Der Heijden AA, Elders PJ, et al. The Association between Social Jetlag, the Metabolic Syndrome, and Type 2 Diabetes Mellitus in the General Population: The New Hoorn Study. Journal of Biological Rhythms. 2017;32(4):359-68.

1. Mcmahon DM, Burch JB, Wirth MD, Youngstedt SD, Hardin JW, Hurley TG, et al. Persistence of social jetlag and sleep disruption in healthy young adults. Chronobiology International. 2017;35(3):312-28.
2. De Medeiros Lopes XF, Araujo MFS, Lira NCC, Dantas DS, De Souza JC. Social, Biological and Behavioral Factors Associated with Social Jet Lag and Sleep Duration in University Students from a Low Urbanized City. J Multidiscip Healthc. 2022;15:11-20.

7 Festing MF. On determining sample size in experiments involving laboratory animals. Lab Anim. 2018;52(4):341-50.

8 Hepler C, Weidemann BJ, Waldeck NJ, Marcheva B, Cedernaes J, Thorne AK, et al. Time-restricted feeding mitigates obesity through adipocyte thermogenesis. Science. 2022;378(6617):276-84.

9 Pivovarova O, Jürchott K, Rudovich N, Hornemann S, Ye L, Möckel S, et al. Changes of dietary fat and carbohydrate content alter central and peripheral clock in humans. J Clin Endocrinol Metab. 2015;100(6):2291-302.

10 Jiang J, Zhang P, Yuan Y, Xu X, Wu T, Zhang Z, et al. Prolactin deficiency drives diabetes-associated cognitive dysfunction by inducing microglia-mediated synaptic loss. J Neuroinflammation. 2024;21(1):295.

11 Hughey JJ, Hastie T, Butte AJ. ZeitZeiger: supervised learning for high-dimensional data from an oscillatory system. Nucleic Acids Res. 2016;44(8):e80.

12 Mortimer T, Welz PS, Benitah SA, Sassone-Corsi P, Koronowski KB. Collecting mouse livers for transcriptome analysis of daily rhythms. STAR Protoc. 2021;2(2):100539.

13 Eckel-Mahan KL, Patel VR, de Mateo S, Orozco-Solis R, Ceglia NJ, Sahar S, et al. Reprogramming of the circadian clock by nutritional challenge. Cell. 2013;155(7):1464-78.

**Table S1** Baseline characteristics of the study population categorized by social jetlag levels

| **Item** | **Control group**  **(SJL < 1 h, *n* = 125)** | **SJL group**  **(SJL ≥ 1 h, *n* = 72)** | ***P-*value** |
| --- | --- | --- | --- |
| Demographic factors |  |  |  |
| MASLD [*n* (%)] | 35 (28.0) | 30 (41.7) | < 0.001 |
| Male/female | 31/94 | 37/35 | < 0.001 |
| Age [year, *M* (*Q*_1_, *Q*_3_)] | 31.0 (26.8, 37.3) | 33.0 (28.0, 39.0) | 0.865 |
| BMI [kg/m^2^, *M* (*Q*_1_, *Q*_3_)] | 28.3 (24.8, 33.0) | 28.6 (23.6, 34.5) | 0.759 |
| SBP [mmHg, *M* (*Q*_1_, *Q*_3_)] | 145.00 (130.00, 156.50) | 145.00 (123.00, 167.00) | 0.355 |
| DBP [mmHg, *M* (*Q*_1_, *Q*_3_)] | 88.50 (80.75, 102.00) | 90.00 (79.00, 100.00) | 0.814 |
| Waist [cm, *M* (*Q*_1_, *Q*_3_)] | 115.50 (109.25, 129.25) | 116.00 (106.00, 134.00) | 0.804 |
| Metabolic parameters [*M* (*Q*_1_, *Q*_3_)] |  |  |  |
| HbA1c (%) | 5.7 (5.3, 6.6) | 5.9 (5.4, 7.5) | 0.005 |
| FBG (mmol/L) | 5.29 (4.70, 6.59) | 5.37 (4.65, 7.43) | 0.006 |
| FINS (mIU/ml) | 21.85 (15.78, 31.93) | 22.90 (14.90, 35.40) | 0.274 |
| HOMA-IR | 4.95 (2.91, 7.06) | 5.66 (3.35, 9.61) | 0.017 |
| ALT (U/L) | 31.90 (22.38, 53.18) | 40.50 (25.70, 60.90) | < 0.001 |
| AST (U/L) | 20.55 (16.95, 32.55) | 27.70 (20.70, 38.20) | < 0.001 |
| TG (mmol/L) | 1.54 (1.10, 2.16) | 1.8 (1.39, 2.24) | 0.066 |
| TC (mmol/L) | 4.73 (4.07, 5.37) | 5.04 (4.63, 5.48) | 0.068 |
| HDL (mmol/L) | 1.04 (0.89, 1.27) | 0.97 (0.91, 1.19) | 0.391 |
| LDL (mmol/L) | 2.86 (2.41, 3.21) | 3.13 (2.75, 3.44) | 0.063 |
| Pituitary hormones and circadian parameters [*M* (*Q*_1_, *Q*_3_)] |  |  |  |
| PRL (μg/L) |  |  |  |
| 8:00 | 11.28 (8.53, 14.59) | 7.48 (5.75, 9.23) | < 0.001 |
| 16:00 | 9.84 (7.47, 14.07) | 6.74 (5.48, 8.80) | < 0.001 |
| 24:00 | 11.29 (8.81, 16.17) | 6.82 (5.58, 7.93) | < 0.001 |
| PRL amplitude | 2.61 (1.68, 4.56) | 1.70 (1.01, 2.41) | < 0.001 |
| PRL phase | -0.04 (-0.69, 0.93) | -0.13 (-0.87, 0.55) | 0.197 |
| ACTH (pmol/L) |  |  |  |
| 8:00 | 3.85 (2.57, 8.27) | 3.73 (2.29, 7.55) | 0.471 |
| 16:00 | 3.87 (2.44, 5.34) | 3.97 (3.11, 5.66) | 0.096 |
| 24:00 | 1.89 (1.23, 3.72) | 2.31 (1.59, 3.93) | 0.905 |
| ACTH amplitude | 2.00 (1.07, 3.39) | 2.12 (1.34, 3.29) | 0.694 |
| ACTH phase | -0.60 (-0.89, 0.60) | -0.71 (-1.05, 0.51) | 0.273 |
| TSH (mIU/L) |  |  |  |
| 8:00 | 2.19 (1.54, 2.98) | 1.66 (1.1, 2.34) | 0.056 |
| 16:00 | 1.49 (1.19, 2.07) | 1.38 (1.16, 1.74) | 0.653 |
| 24:00 | 2.04 (1.49, 3.10) | 1.92 (1.53, 2.60) | 0.797 |
| TSH amplitude | 0.60 (0.37, 1.04) | 0.54 (0.32, 1.00) | 0.353 |
| TSH phase | -0.17 (-0.79, 0.93) | 0.57 (-0.62, 1.12) | 0.323 |
| LH (mIU/L) |  |  |  |
| 8:00 | 3.78 (2.80, 6.32) | 4.12 (2.79, 5.6) | 0.799 |
| 16:00 | 4.35 (3.15, 6.76) | 4.4 (3.76, 5.36) | 0.721 |
| 24:00 | 3.74 (2.33, 5.45) | 4.45 (2.80, 5.88) | 0.407 |
| LH amplitude | 1.09 (0.64, 2.03) | 0.93 (0.55, 1.60) | 0.239 |
| LH phase | -0.3 (-1.17, 0.81) | 0.17 (-1.14, 0.97) | 0.454 |
| FSH (mIU/L) |  |  |  |
| 8:00 | 5.88 (3.9, 7.59) | 5.42 (3.88, 7.45) | 0.452 |
| 16:00 | 6.01 (3.99, 7.80) | 5.46 (3.78, 7.55) | 0.837 |
| 24:00 | 5.53 (4.10, 7.20) | 5.78 (4.18, 7.76) | 0.687 |
| FSH amplitude | 0.53 (0.33, 0.96) | 0.64 (0.29, 0.87) | 0.390 |
| FSH phase | -0.16 (-0.62, 0.50) | -0.57 (-1.04, 0.21) | 0.062 |
| GH (μg/L) |  |  |  |
| 8:00 | 0.06 (0.05, 0.17) | 0.06 (0.05, 0.14) | 0.156 |
| 16:00 | 0.12 (0.07, 0.31) | 0.15 (0.05, 0.27) | 0.797 |
| 24:00 | 0.29 (0.13, 0.74) | 0.28 (0.14, 0.84) | 0.862 |
| GH amplitude | 0.22 (0.08, 0.68) | 0.13 (0.06, 0.69) | 0.478 |
| GH phase | -0.68 (-1.53, 0.07) | -0.43 (-1.46, 0.68) | 0.344 |

*MASLD* metabolic dysfunction-associated steatotic liver disease, *BMI* body mass index, *SBP* systolic blood pressure, *DBP* diastolic blood pressure, *HbA1c* hemoglobin A1c, *FBG* fasting blood glucose, *FINS* fasting insulin, *HOMA-IR* homeostasis model assessment of insulin resistance, *ALT* alanine aminotransferase, *AST* aspartate aminotransferase, *TG* triglycerides, *TC* total cholesterol, *HDL* high-density lipoprotein cholesterol, *LDL* low-density lipoprotein cholesterol, *PRL* prolactin, *ACTH* adrenocorticotropic hormone, *TSH* thyrotropin-stimulating hormone, *LH* luteinizing hormone, *FSH* follicle-stimulating hormone, *GH* growth hormone

**Table S2** SJL profiles of the study population with and without MASLD [*n* (%)]

| **SJL (h)** | **Non-MASLD (*n* = 132)** | **MASLD (*n* = 65)** | ***P*-value** |
| --- | --- | --- | --- |
| 0 – 60 | 90 (68.2) | 35 (53.8) | 0.013 |
| 60 – 120 | 22 (16.7) | 6 (9.2) |  |
| 120 – 180 | 12 (9.1) | 11 (16.9) |  |
| ≥ 180 | 8 (6.1) | 13 (20.0) |  |

*SJL* social jetlag, *MASLD* metabolic dysfunction-associated steatotic liver disease

**Table S3** Primers used for qRT-PCR assays

| **Gene name** | **Forward primer (5’** – **3’)** | **Reverse primer (5’** – **3’)** |
| --- | --- | --- |
| h-*β-actin* | GCGGGAAATCGTGCGTGAC | CAGGAAGGAAGGCTGGAAGAGTG |
| m-*β-actin* | GTGACGTTGACATCCGTAAAGA | GCCGGACTCATCGTACTCC |
| r-*β-actin* | TCAGGTCATCACTATCGGCAA | AGCACTGTGTTGGCATAGAGG |
| m-*Ccnd1* | GCGTACCCTGACACCAATCTC | CTCCTCTTCGCACTTCTGCTC |
| m-*Tnfrsf11a* | ACCATTTCCCGACCCAGAGA | CCTCAGTCGGGATCAGTGTG |
| m-*Slc2a2* | CCTTGGGCCTTACGTGTTCT | CACACTCTCTGAAGACGCCA |
| m-*Sos2* | ATTCCACGCAGCTGTCACTT | TGGTGGCAGCTTTGGTAAGA |
| m-*Mapk12* | AGTGGCTTTTACCGCCAGG | GACTGGAAGGGCCGATACAG |
| h-*Acc* | ATGTCTGGCTTGCACCTAGTA | CCCCAAAGCGAGTAACAAATTCT |
| h-*Fas* | AAGGACCTGTCTAGGTTTGATGC | TGGCTTCATAGGTGACTTCCA |
| m-*Acc* | CTTCCTGACAAACGAGTCTGG | CTGCCGAAACATCTCTGGGA |
| m-*Fasn* | GGAGGTGGTGATAGCCGGTAT | TGGGTAATCCATAGAGCCCAG |
| m-*Rorc* | GACCCACACCTCACAAATTGA | AGTAGGCCACATTACACTGCT |
| m-*Rorα* | GTGGAGACAAATCGTCAGGAAT | TGGTCCGATCAATCAAACAGTTC |
| m-*Reverbα* | TGAACGCAGGAGGTGTGATTG | GAGGACTGGAAGCTATTCTCAGA |
| h-*Rorα* | ACTCCTGTCCTCGTCAGAAGA | CATCCCTACGGCAAGGCATTT |
| h-*Reverbα* | TGGACTCCAACAACAACACAG | GATGGTGGGAAGTAGGTGGG |
| m-*Reverbβ* | TTTAGTGGCATGGTTCTACTGTG | AGCCTTCGCAAGCATGAACT |
| r-*Rorα* | GAGACAAATCGTCAGGAATCCAT | CCACAGCCAGGCACTTCTG |
| r-*Reverbα* | GGTGCCTAGAATCCTGATTGTGA | TCCGCTGGAGCCAATGTAG |

*Ccnd1* cyclin D1, *Tnfrsf11a* tumor necrosis factor super family member 11a, *Slc2a2* solute carrier 2a2, *Sos2* SOS Ras/Rho guanine nucleotide exchange factor 2, *Mapk12* mitogen-activated protein kinase 12, *Acc* acetyl coenzyme A carboxylasel, *Fasn* fatty acid synthase, *Rorc* retinoic acid receptor related orphan receptor C, *Rorα* retinoic acid receptor related orphan receptor α, *Reverbα* nuclear receptor subfamily 1 group D member 1

**
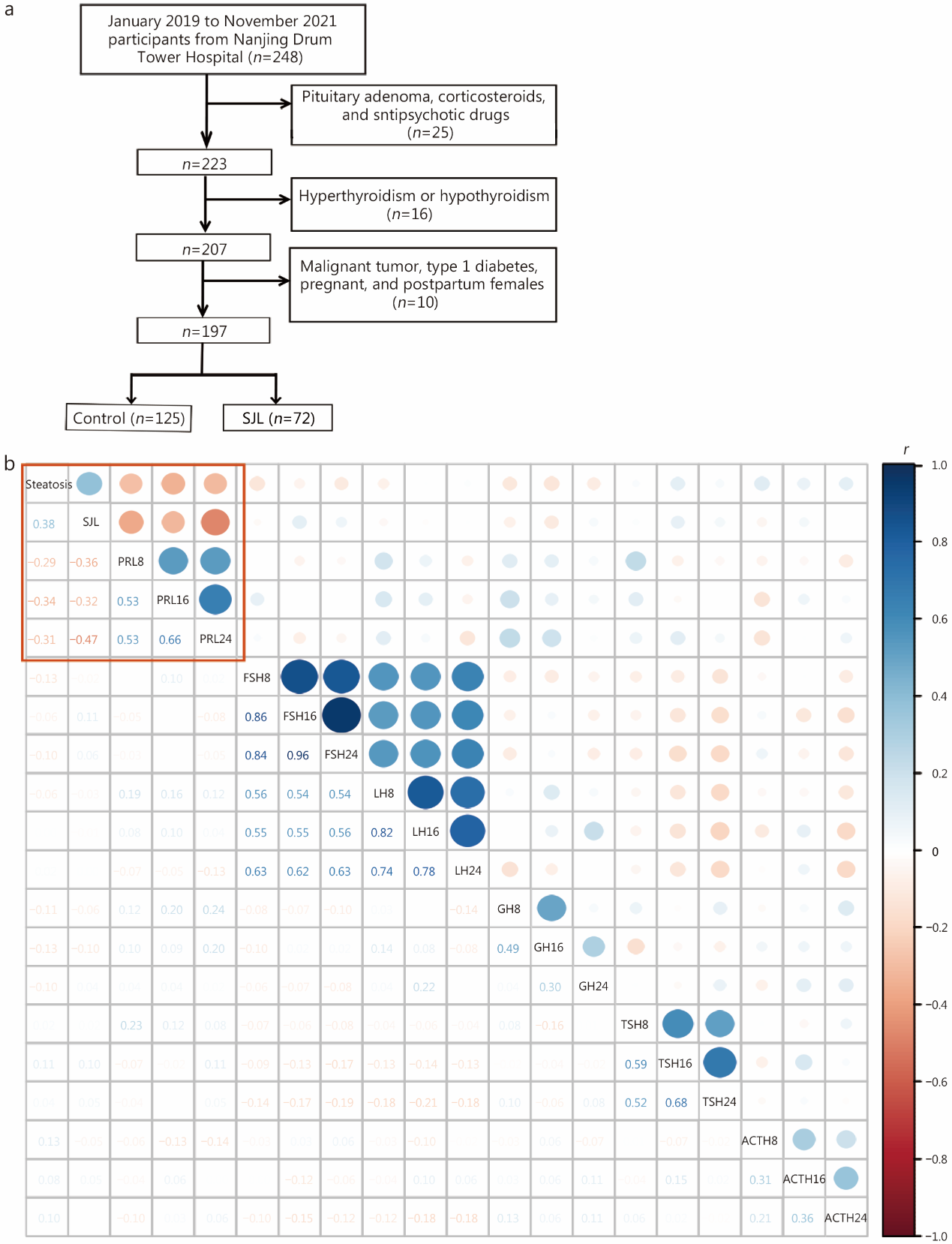
**

**Fig. S1** Illustration of the flowchart of the study population (**a**) and the correlation between jetlag and pituitary hormones and steatosis severity (**b**). Heatmap displaying Spearman correlation coefficients between social jetlag (SJL) values and pituitary hormones at different time points (8:00, 16:00, 24:00) and steatosis severity. PRL prolactin, ACTH adrenocorticotropic hormone, TSH thyrotropin-stimulating hormone, LH luteinizing hormone, FSH follicle-stimulating hormone, GH growth hormone


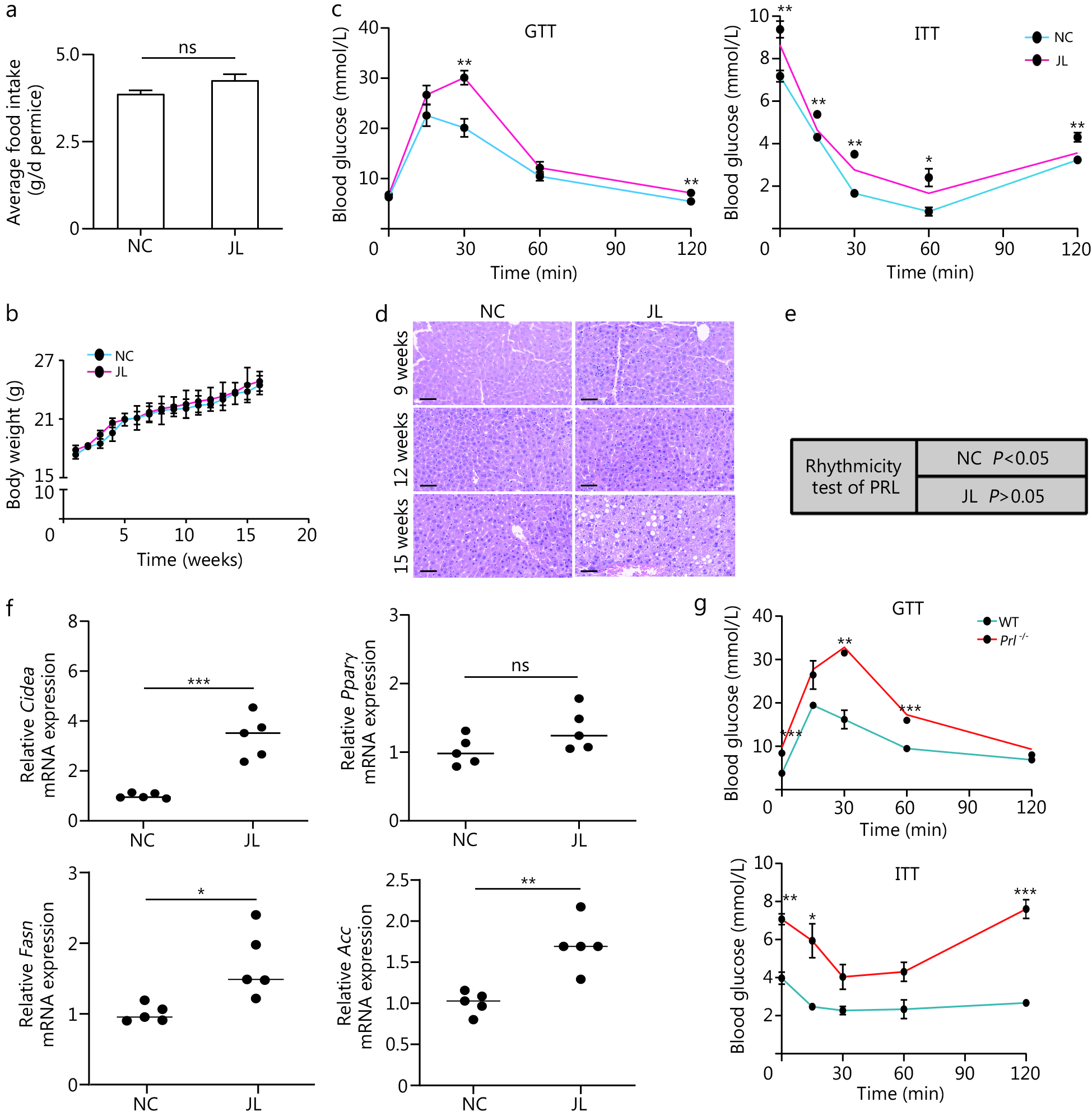


**Fig. S2** The effect of social jetlag (SJL) on energy homeostasis. **a** Average food intake of mice under normal light cycle (NC) and jetlag (JL) during 16 weeks (*n* = 5). **b** Body weight of mice under NC and JL. **c** Glucose levels of glucose tolerance test (GTT) and insulin tolerance test (ITT) of mice under NC and JL (*n* = 5). **d** H&E staining of the liver in mice at 9, 12, and 15 weeks of NC and JL. Scale bar = 50 μm. **e** Rhythmicity test of serum PRL levels in NC and JL mice, the test was completed using the CircaCompare algorithm. **f** qRT-PCR analysis of mRNA levels of hepatic genes involved in lipid metabolism in normal light cycle (NC) and jetlag (JL) mice, data were normalized to *β-actin* mRNA levels (*n* = 5). **g** Glucose levels of GTT and ITT of wild-type (WT) and *Prl^-/-^* mice (*n* = 3). ^*^*P* < 0.05, ^**^*P* < 0.01, ^***^*P* < 0.001, ns not significant. *P*-values were calculated by independent-sample *t*-test. Cidea cell death-inducing DNA fragmentation factor-α-like effector A, Pparγ peroxisome proliferator-activated receptor γ, Fasn fatty acid synthase, Acc acetyl coenzyme A carboxylasel

**
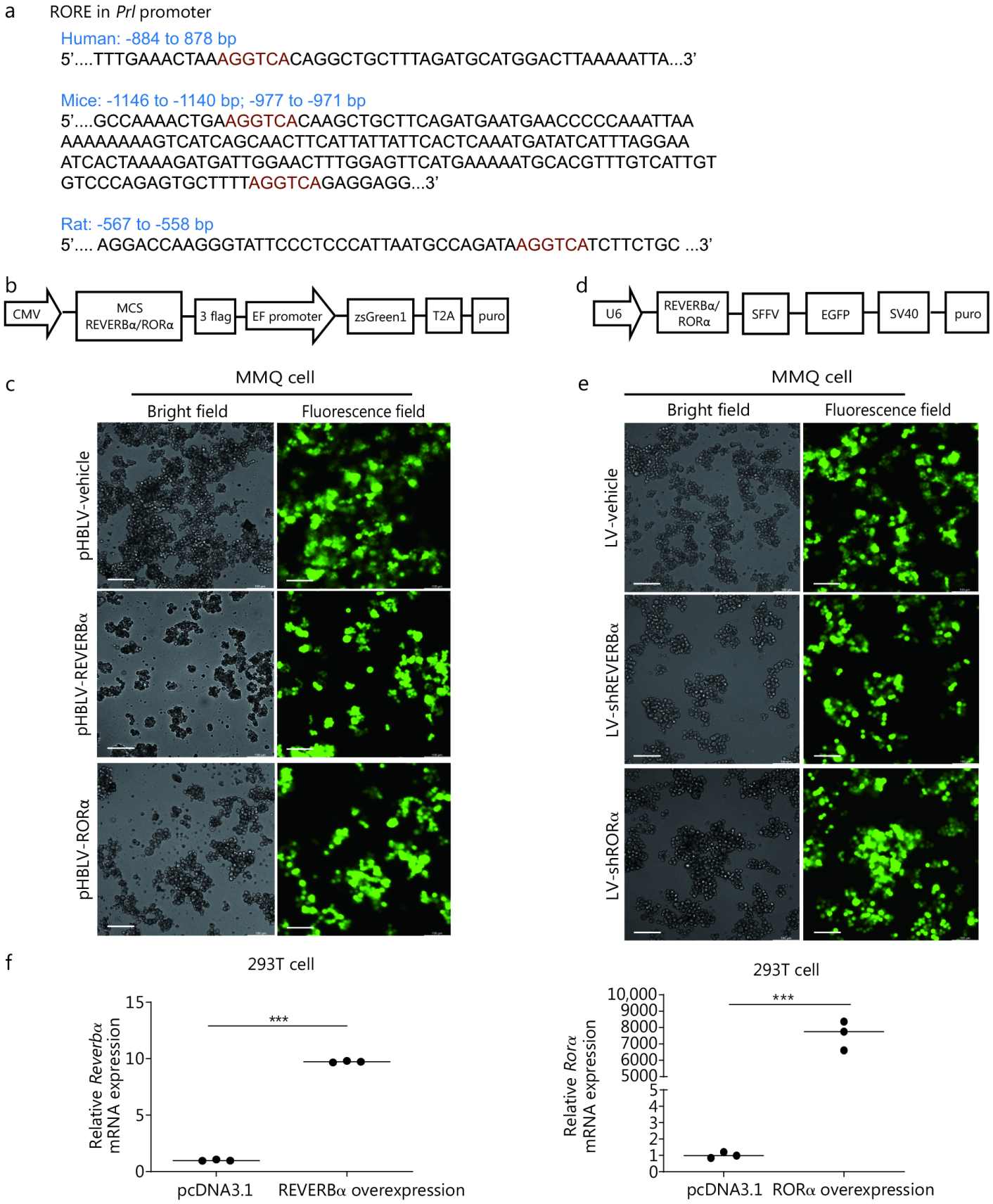
**

**Fig. S3** Overexpression and knockdown of *Reverbα* and *Rorα* in vitro. **a** Analysis of the predicted amino acid sequence of the *Prl* promoter in humans, mice, and rats. **b** Diagram showing the constructed vectors pHBLV. **c** Bright field and fluorescence of MMQ cell line transfected with pHBLV-vehicle, pHBLV-REVERBα, and pHBLV-RORα. **d** Diagram showing the constructed vectors of lentivirus with shRNA. **e** Bright field and fluorescence MMQ cell line transfected with LV-vehicle, LV-REVERBα, and LV-RORα. **f** mRNA levels of *Reverbα* and *Rora* in 293T cells that overexpressed REVERBα and RORα, as determined by qRT-PCR analysis, data were normalized to *β-actin* mRNA levels, data are expressed as the mean ± SEM. *^***^P* < 0.001. *P*-values were calculated by independent-sample *t*-test. Scale bar = 50 μm. Reverbα nuclear receptor subfamily 1 group D member 1, Rorα retinoic acid receptor-related orphan receptor α, CMV cytomegalovirus, MCS multi-clone site, EF elongation factor, T2A 2A-like cis-acting hydrolase elements of Thosea asigna virus, SFFV spleen focus-forming virus, EGFP enhanced green fluorescent protein, SV40 simian virus 40

**
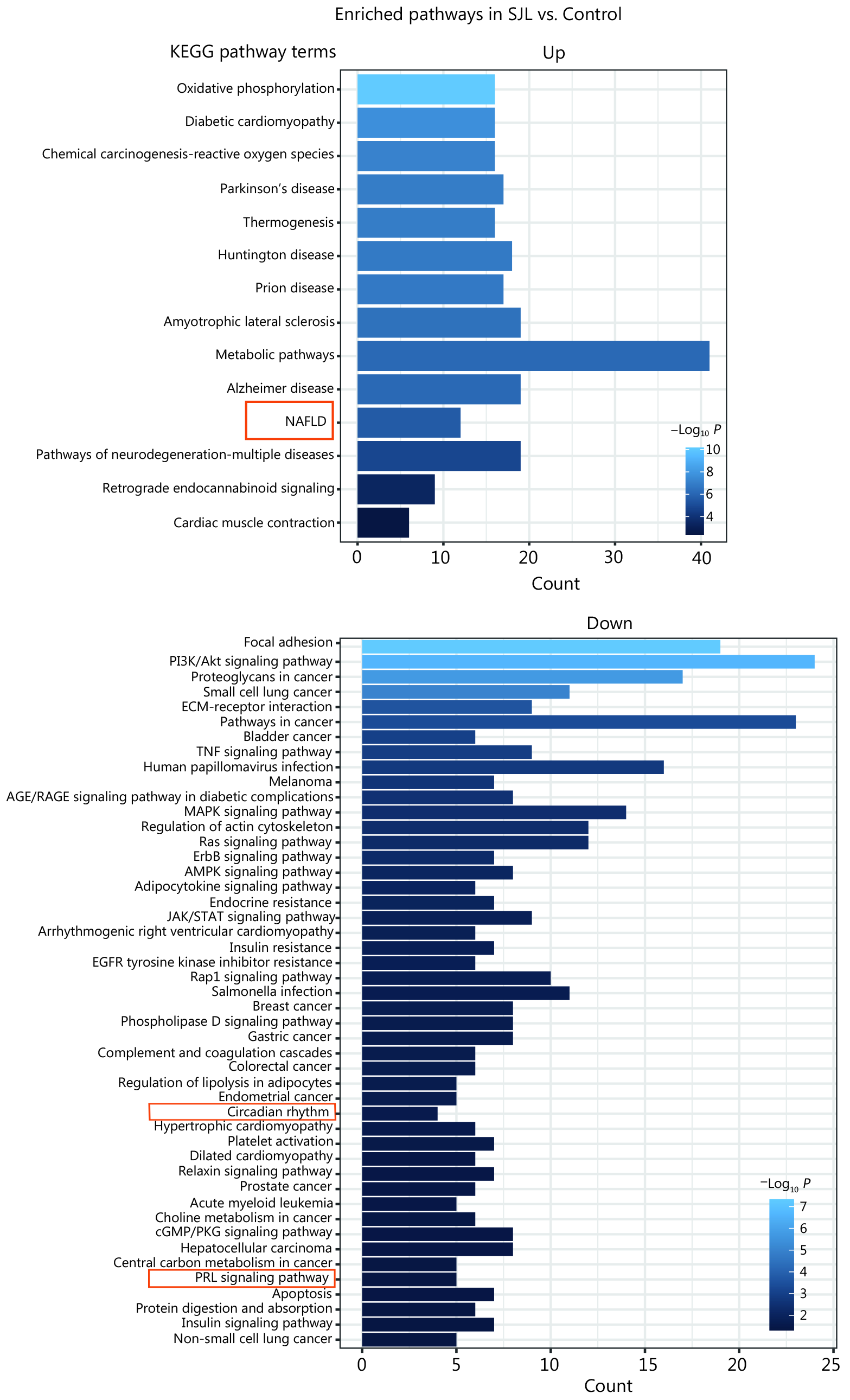
**

**Fig. S4** Kyoto Encyclopedia of Genes and Genomes (KEGG) enrichment pathways based on up- and down-regulated DEGs from SJL vs. control subjects. NAFLD non-alcoholic fatty liver disease, PI3K phosphatidylinositol 3-kinase, Akt protein kinase B, ECM extracellular matrix, TNF tumor necrosis factor, AGE/RAGE receptor for advanced-glycation end products, MAPK mitogen-activated protein kinase, ErbB receptor of epidermal growth factor receptor, AMPK AMP-activated protein kinase, JAK janus kinase, STAT signal transducer and activator of transcription, PRL prolacin, EGFR epidermal growth factor receptor, cGMP/PKG cyclic guanosine monophosphate/protein kinase G


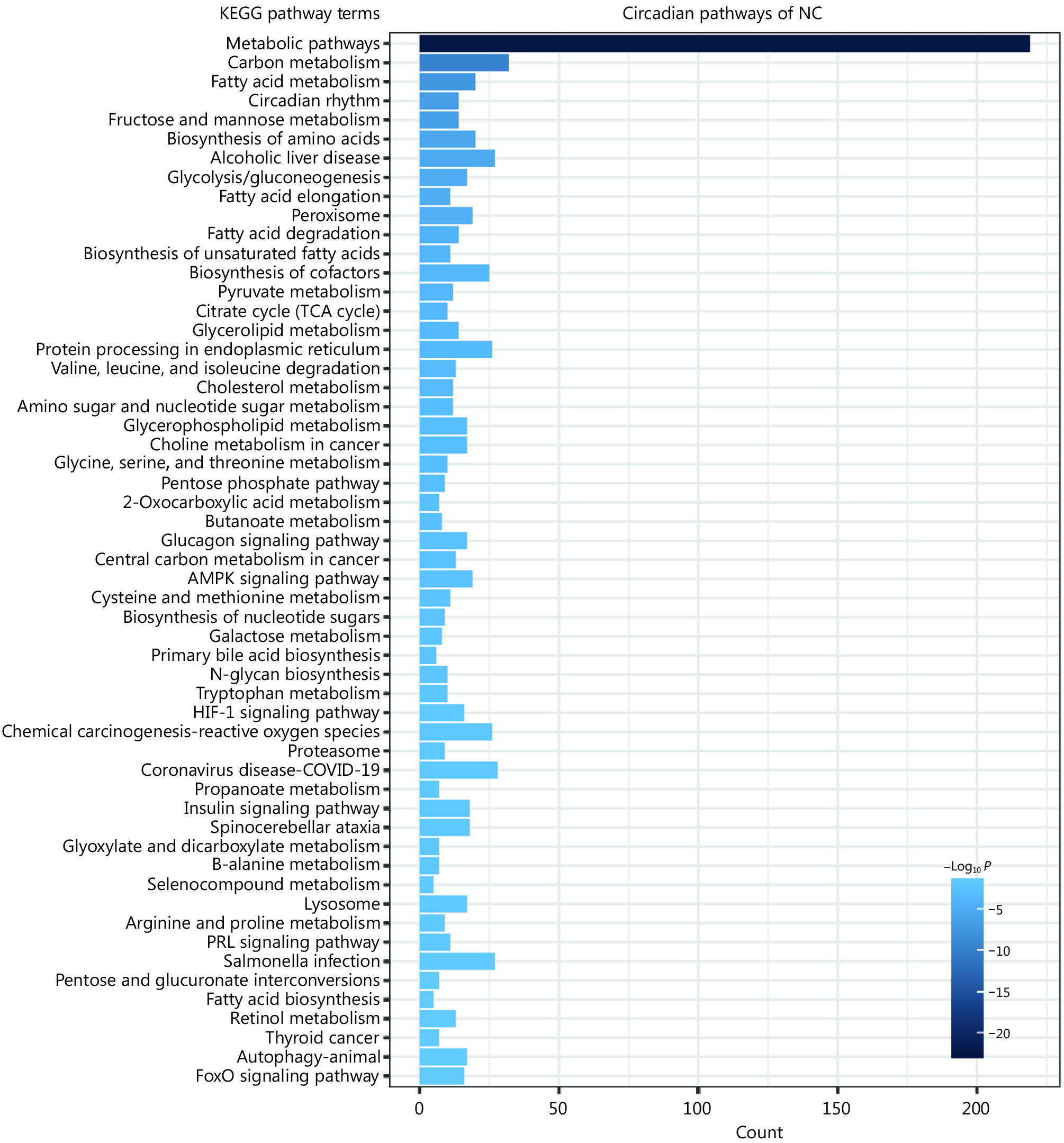


**Fig. S5** Kyoto Encyclopedia of Genes and Genomes (KEGG) enrichment pathways of circadian genes in the liver of female mice under normal light cycle (NC) (*n* = 3 per time point, rhythmicity was tested via JTK_cycle algorithm and *P* < 0.05 was considered as significant rhythmic). TCA tricarboxylic acid, AMPK AMP-activated protein kinase, COVID-19 coronavirus disease 2019, HIF hypoxia-inducible factor, FoxO forkhead box O


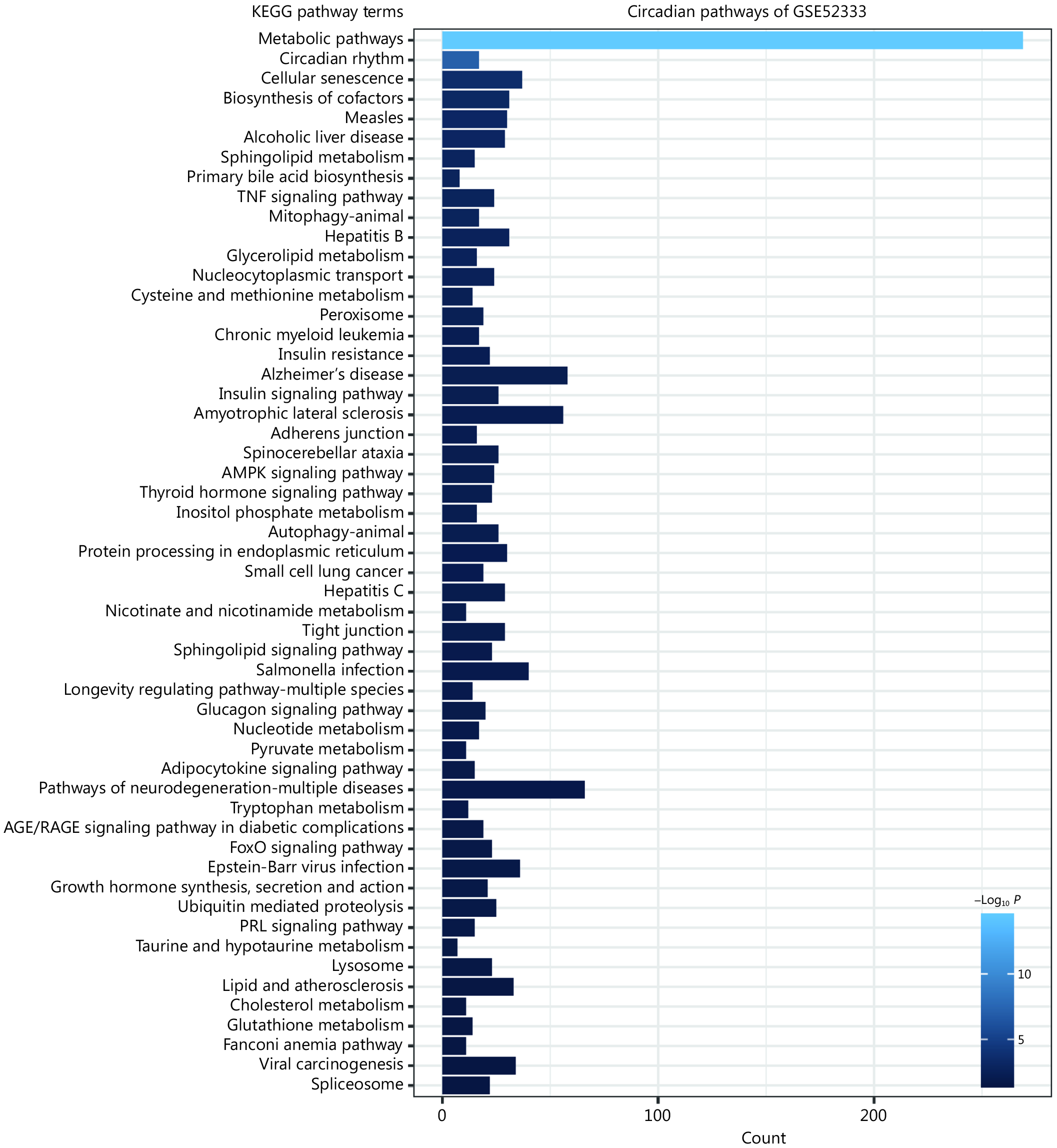


**Fig. S6** Kyoto Encyclopedia of Genes and Genomes (KEGG) enrichment of circadian genes using microarray data in male mice under normal light cycle retrieved from public Gene Expression Omnibus (GEO) database (*n* = 3 per time point, rhythmicity was tested via JTK_cycle algorithm and *P* < 0.05 was considered as significant rhythmic). TNF tumor necrosis factor, AMPK AMP-activated protein kinase, AGE/RAGE receptor for advanced-glycation end products, FoxO forkhead box O, PRL prolacin


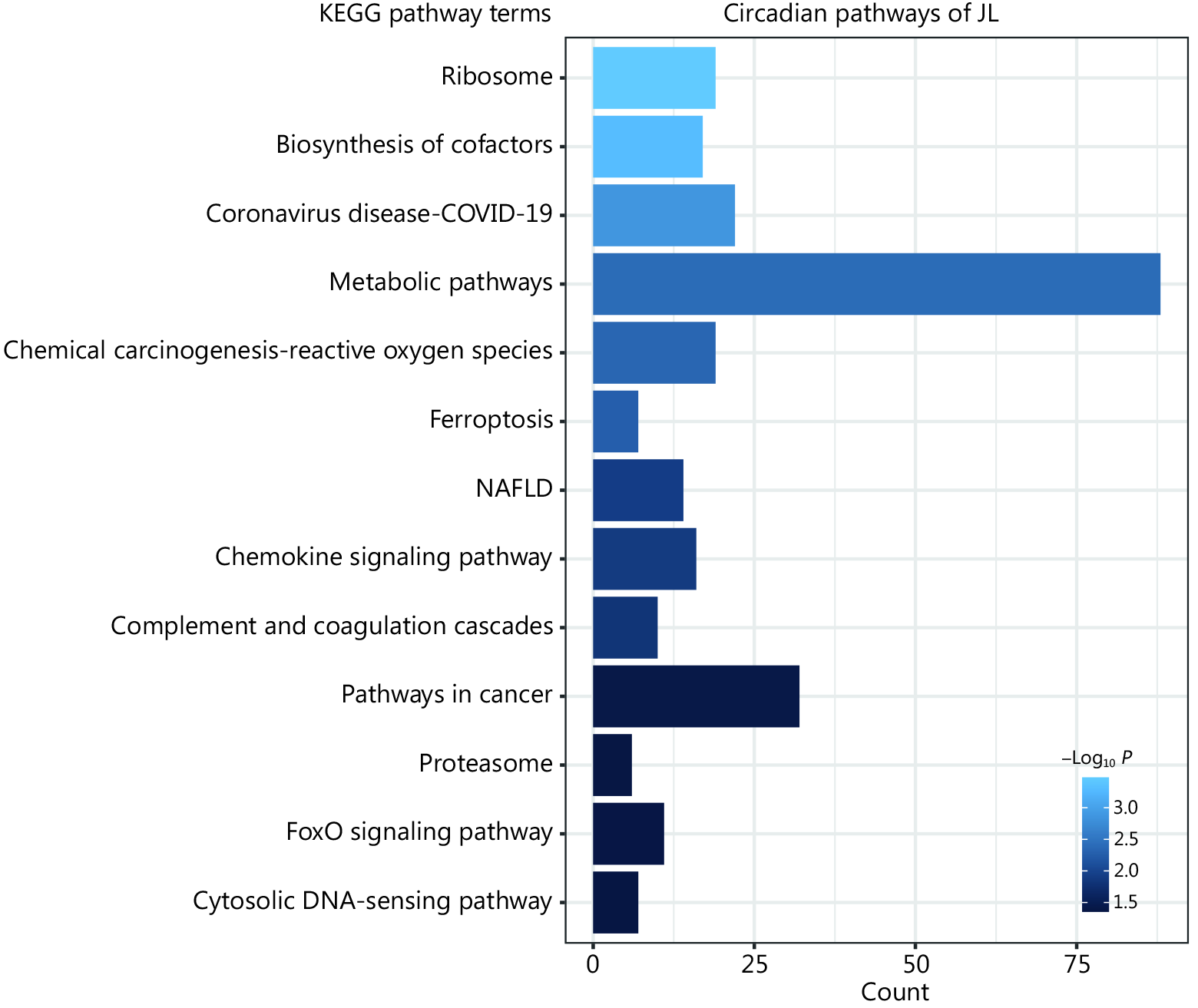


**Fig. S7** Kyoto Encyclopedia of Genes and Genomes (KEGG) enrichment pathways of circadian genes in the liver of female mice under jetlag (JL) (*n* = 3 per time point, rhythmicity was tested via JTK_cycle algorithm and *P* < 0.05 was considered as significant rhythmic). COVID-19 coronavirus disease 2019, NAFLD non-alcoholic fatty liver disease, FoxO forkhead box O


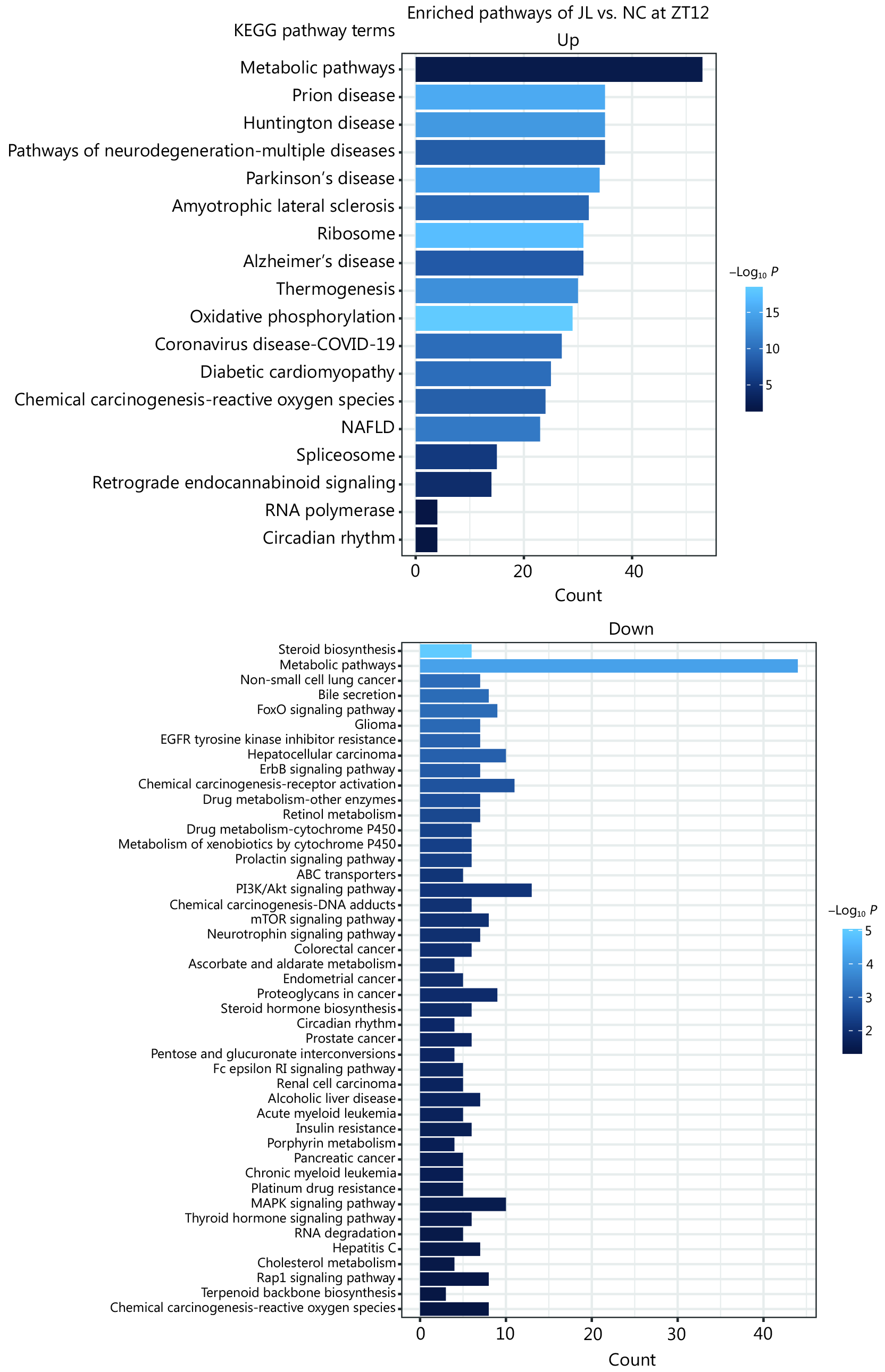


**Fig. S8** Jetlag (JL) altered hepatic transcriptome of mice at ZT12. Kyoto Encyclopedia of Genes and Genomes (KEGG) enrichment pathways of up-regulated genes and down-regulated of female mice under normal light cycle (NC; *n* = 3) and JL (*n* = 3) at ZT12, the threshold of differentially expressed genes (DEGs) is log_2_ fold change (FC) is 1 and adjusted *P* < 0.05. COVID-19 coronavirus disease 2019, NAFLD non-alcoholic fatty liver disease, FoxO forkhead box O, EGFR epidermal growth factor receptor, ErbB receptor of epidermal growth factor receptor, ABC ATP-binding cassette, PI3K/Akt phosphoinositide 3-kinases-serine/threonine kinase 1, mTOR mechanistic target of rapamycin kinase, MAPK mitogen-activated protein kinase, Rap1 ras-related protein 1


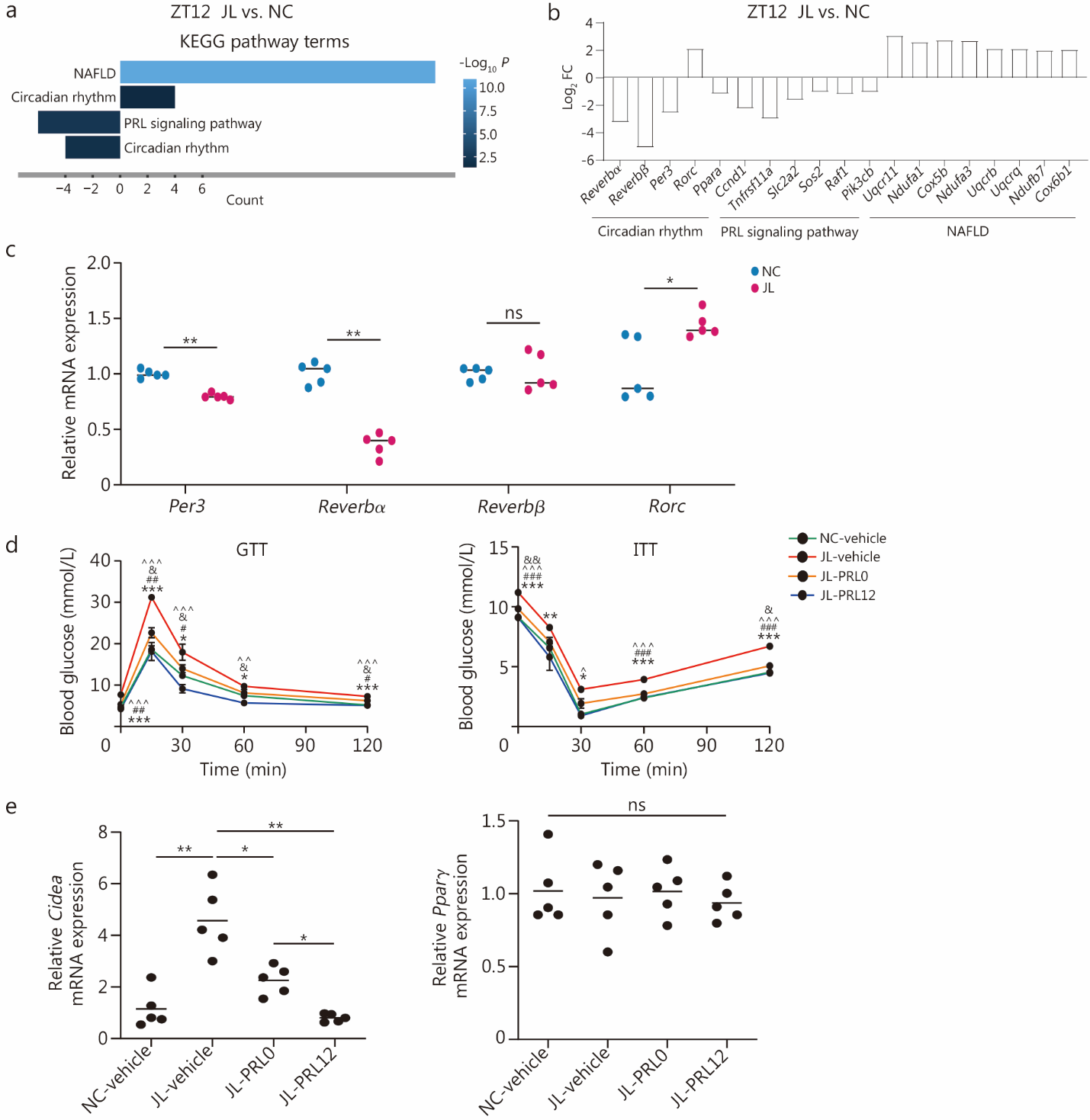


**Fig. S9** Expression of differentially expressed genes between normal light cycle (NC) and jetlag (JL) group at ZT12 and the effect of prolactin (PRL) treatment. **a** Kyoto Encyclopedia of Genes and Genomes (KEGG) analyses of up-regulated and down-regulated genes in indicated pathways. **b** Log_2_ fold change (FC) of annotated differentially expressed genes (DEGs) enriched in indicated pathways. **c** qRT-PCR validation of mRNA levels of genes involved in circadian rhythm processes identified from RNA-seq between NC and JL group at ZT12 (*n* = 5). Data were normalized to *β-actin* mRNA levels. **d** Glucose levels of glucose tolerance test (GTT) and insulin tolerance test (ITT) of mice in mice from NC-vehicle, JL-vehicle, JL-PRL0, and JL-PRL12 (*n* = 3). ^*^*P* < 0.05, ^**^*P* < 0.01, ^**^*P* < 0.001 between JL-vehicle and NC-vehicle; ^#^*P* < 0.05, ^##^*P* < 0.01, ^###^*P* < 0.001 between JL-PRL0 and JL-vehicle; ^&^*P* < 0.05, ^&&^*P* < 0.01, ^&&&^*P* < 0.001 between JL-PRL12 and JL-PRL0; ^^^*P* < 0.05, ^^^^*P* < 0.01, ^^^^^*P* < 0.001 between JL-PRL12 and JL-vehicle. **e** Hepatic genes involved in lipid metabolism in NC and JL mice (*n* = 5). Data were normalized to *β-actin* mRNA levels. ^*^*P* < 0.05, ^**^*P* < 0.01, ^**^*P* < 0.001, ns not significant. *P*-values were calculated by independent-sample *t-*test (**c**) and one-way ANOVA (**d, e**). NAFLD non-alcoholic fatty liver disease, Cidea cell death-inducing DNA fragmentation factor-α-like effector A, Pparγ peroxisome proliferator-activated receptor γ, Per3 period circadian regulator 3, Reverbα Nuclear receptor subfamily 1 group D member 1, Reverbβ Nuclear receptor subfamily 1 group D member 2, Rorc retinoic acid receptor-related orphan receptor
